# Phase variable colony variants are conserved across *Gardnerella* spp. and exhibit different virulence-associated phenotypes

**DOI:** 10.1128/msphere.00450-24

**Published:** 2024-06-27

**Authors:** Erin M. Garcia, Amy K. Klimowicz, Laahirie Edupuganti, Madeline A. Topf, Shraddha R. Bhide, Dawson J. Slusser, Samantha M. Leib, Cayden L. Coddington, Andrey Matveyev, Gregory A. Buck, Kimberly K. Jefferson, Caitlin S. Pepperell, Joseph P. Dillard

**Affiliations:** 1 Department of Medical Microbiology and Immunology, University of Wisconsin-Madison, Madison, Wisconsin, USA; 2 Center for Microbiome Engineering and Data Analysis, Virginia Commonwealth University, Richmond, Virginia, USA; 3 Department of Microbiology and Immunology, School of Medicine, Virginia Commonwealth University, Richmond, Virginia, USA; 4 Department of Medicine, Division of Infectious Diseases, University of Wisconsin-Madison, Madison, Wisconsin, USA; University of Kentucky College of Medicine, Lexington, Kentucky, USA

**Keywords:** phase variation, *Gardnerella*, phenotypic variation, vaginosis

## Abstract

The *Gardnerella* genus, comprising at least 13 species, is associated with the polymicrobial disorder bacterial vaginosis (BV). However, the details of BV pathogenesis are poorly defined, and the contributions made by individual species, including *Gardnerella* spp., are largely unknown. We report here that colony phenotypes characterized by size (large and small) and opacity (opaque and translucent) are phase variable and are conserved among all tested *Gardnerella* strains, representing at least 10 different species. With the hypothesis that these different variants could be an important missing piece to the enigma of how BV develops *in vivo*, we characterized their phenotypic, proteomic, and genomic differences. Beyond increased colony size, large colony variants showed reduced vaginolysin secretion and faster growth rate relative to small colony variants. The ability to inhibit the growth of *Neisseria gonorrhoeae* and commensal *Lactobacillus* species varied by strain and, in some instances, differed between variants. Proteomics analyses indicated that 127–173 proteins were differentially expressed between variants. Proteins with increased expression in large variants of both strains were associated with amino acid and protein synthesis and protein folding, whereas those increased in small variants were related to nucleotide synthesis, phosphate transport, ABC transport, and glycogen breakdown. Furthermore, whole genome sequencing analyses revealed an abundance of genes associated with variable homopolymer tracts, implicating slipped strand mispairing in *Gardnerella* phase variation and illuminating the potential for previously unrecognized heterogeneity within clonal populations. Collectively, these results suggest that phase variants may be primed to serve different roles in BV pathogenesis.

**IMPORTANCE:**

Bacterial vaginosis is the most common gynecological disorder in women of childbearing age. *Gardnerella* species are crucial to the development of this dysbiosis, but the mechanisms involved in the infection are not understood. We discovered that *Gardnerella* species vary between two different forms, reflected in bacterial colony size. A slow-growing form makes large amounts of the toxin vaginolysin and is better able to survive in human cervix tissue. A fast-growing form is likely the one that proliferates to high numbers just prior to symptom onset and forms the biofilm that serves as a scaffold for multiple BV-associated anaerobic bacteria. Identification of the proteins that vary between different forms of the bacteria as well as those that vary randomly provides insight into the factors important for *Gardnerella* infection and immune avoidance.

## INTRODUCTION

*Gardnerella* spp. are Gram-positive actinobacteria that are residents of the human urogenital tract (1). Numerous lines of evidence indicate that *Gardnerella* play a key role in bacterial vaginosis (BV), a dysbiosis in which protective *Lactobacillus* species are replaced by a consortium of anaerobes (2). This condition ultimately reduces the barrier integrity of the vaginal epithelium and increases susceptibility to other urogenital pathogens (3–7). Patients with BV are predisposed to adverse pregnancy outcomes including amnionitis, postpartum endometritis, and premature delivery, the leading cause of neonatal mortality (8, 9). It is hypothesized that *Gardnerella* serve as primary colonizers of the vaginal mucosa, and they increase in relative abundance 3–4 days prior to the onset of symptomatic BV (7, 10). It is also thought that *Gardnerella* dampen the host immune response and establish the BV biofilm prerequisite to the arrival of secondary colonizers, such as *Fannyhessea vaginae* and *Sneathia* spp. (5, 11–13). Strides have been made in understanding the functions of presumed virulence factors including the mucin-degrading sialidases, the pore-forming toxin vaginolysin, and glycogen-degrading enzymes, among others (13–17). However, the mechanistic details of *Gardnerella* pathogenesis remain poorly characterized, partly due to the lack of methods for efficient directed mutagenesis in *Gardnerella* spp. and also due to substantial genetic and phenotypic heterogeneity within the genus (18–21).

*Gardnerella* taxonomy has varied since the discovery of the genus in the 1950s and continues to be a work in progress. Initially characterized as a singular species, later studies consistently supported the division of the *Gardnerella* genus into at least four distinct subgroups, distinguished by differences in the 16S rRNA V6 region or in the gene for the universal 60 kDa chaperonin protein (*cpn60*) (22, 23). Each subgroup exhibits unique genome sizes, GC (guanine plus cytosine) content, and gene content in addition to notable phenotypic differences (19, 24). Most recently, a regrouping of the *Gardnerella* genus into multiple (13+) species was formally proposed following whole genome sequencing of 81 strains and MALDI-TOF (matrix-assisted laser desorption ionization–time of flight) MS (mass spectrometry) analysis of 10 strains (25). Subsequent reports indicate that representatives from multiple species are usually present simultaneously during BV and that the distribution of these representatives is heterogeneous between individuals (26–28). Although some connections have been made between the virulence potential of specific subgroups/species and their association with BV, the details of their unique contributions within the vaginal microbiome are currently unknown (24, 27, 29, 30). This lack of clarity in *Gardnerella* taxonomy has led to difficulties in interpreting the results of previous studies that did not differentiate the species well and further complicates the characterization of host-pathogen interplay during BV. Therefore, in this study, we elected to include multiple species in our analyses.

Although *Gardnerella* heterogeneity at the “community” level in BV is now being explored in more detail, the potential for clonal heterogeneity in *Gardnerella* has yet to be investigated. Phenotypic heterogeneity during infection has important implications for infection outcomes as cell-to-cell differences can alter both how the immune system responds and whether the group as a whole persists. Such diversity can be advantageous, increasing the odds of survival in stable and unstable environments (31). Accordingly, there are multiple mechanisms to generate phenotypic variation within isogenic bacterial populations. Phase variation, wherein bacteria reversibly switch the production of a certain factor “ON” or “OFF,” is one strategy frequently employed by pathogenic organisms (32–37). One of the most common mechanisms is slipped-strand mispairing, resulting in the gain or loss of a short, repeated sequence during DNA replication. These repetitive sequences, either homo- or hetero-polymeric tracts of nucleotides, within or upstream of a gene, ultimately alter the reading frame or lead to altered transcription or translation of a gene or operon (38). This ability to flip a molecular switch helps bacteria survive environmental shifts and to also escape recognition by the host immune response (36). For example, in *Neisseria gonorrhoeae*, phase variation controls the production of Opa proteins. Gain or loss of a five base pair repeat in the coding sequence determines whether translation of the full-length sequence occurs and Opa protein is made or translation is terminated early due to a frameshift (39). The differential expression of these surface proteins, encoded by up to 11 different genes, results in different abilities of the bacteria to bind CEACAM (carcinoembryonic antigen-related cell adhesion molecules) proteins or heparan sulfate proteoglycan on host cells and affects the ability to survive interactions with neutrophils (40–42). Differences in gross colony morphology (small vs large, rough vs smooth, opaque vs translucent) are often an indicator of a phase variable phenotype and have been used to distinguish factor-producing and factor-deficient variants in numerous bacterial species (43–45). Variation of Opa protein expression in *N. gonorrhoeae* directs the assembly of bacterial cells into opaque (Opa^+^) or transparent (Opa^−^) colonies.

In this study, we describe variations in colony morphology that are conserved among multiple species of *Gardnerella*. Colony size and opacity were found to be phase variable, allowing us to distinguish and purify colony variants for characterization. *In vitro* assays performed with variants demonstrated distinct phenotypes with respect to growth, virulence factor production, and interactions with other bacteria. Proteomics analysis identified differences in proteomic profiles, with 127–173 proteins differentially expressed between variants. Ultimately, the differences observed in variants suggest that each may be better adapted to survive under different environmental conditions, and this variation may serve as a bet-hedging strategy to promote survival within the host. We hypothesize that these different variants could be an important missing piece to the puzzle of how BV develops *in vivo*. Furthermore, whole genome sequencing analyses showed an abundance of genes associated with homopolymer tracts, implicating slipped-strand mispairing in *Gardnerella* phase variation, and illuminating the potential for previously unrecognized heterogeneity within clonal populations. Through continued characterization of phenotypic differences and the mechanisms that drive them, we may gain a better understanding of how *Gardnerella* spp. contribute to vaginal dysbiosis and identify novel targets to impede BV development. Addressing diversity within clonal populations, as well as across strains, species, and multi-species communities, will be vital for the rational design of improved BV therapeutics.

## RESULTS

### *Gardnerella* spp. exhibit colony size variants that are phase variable

**Fig 1:**
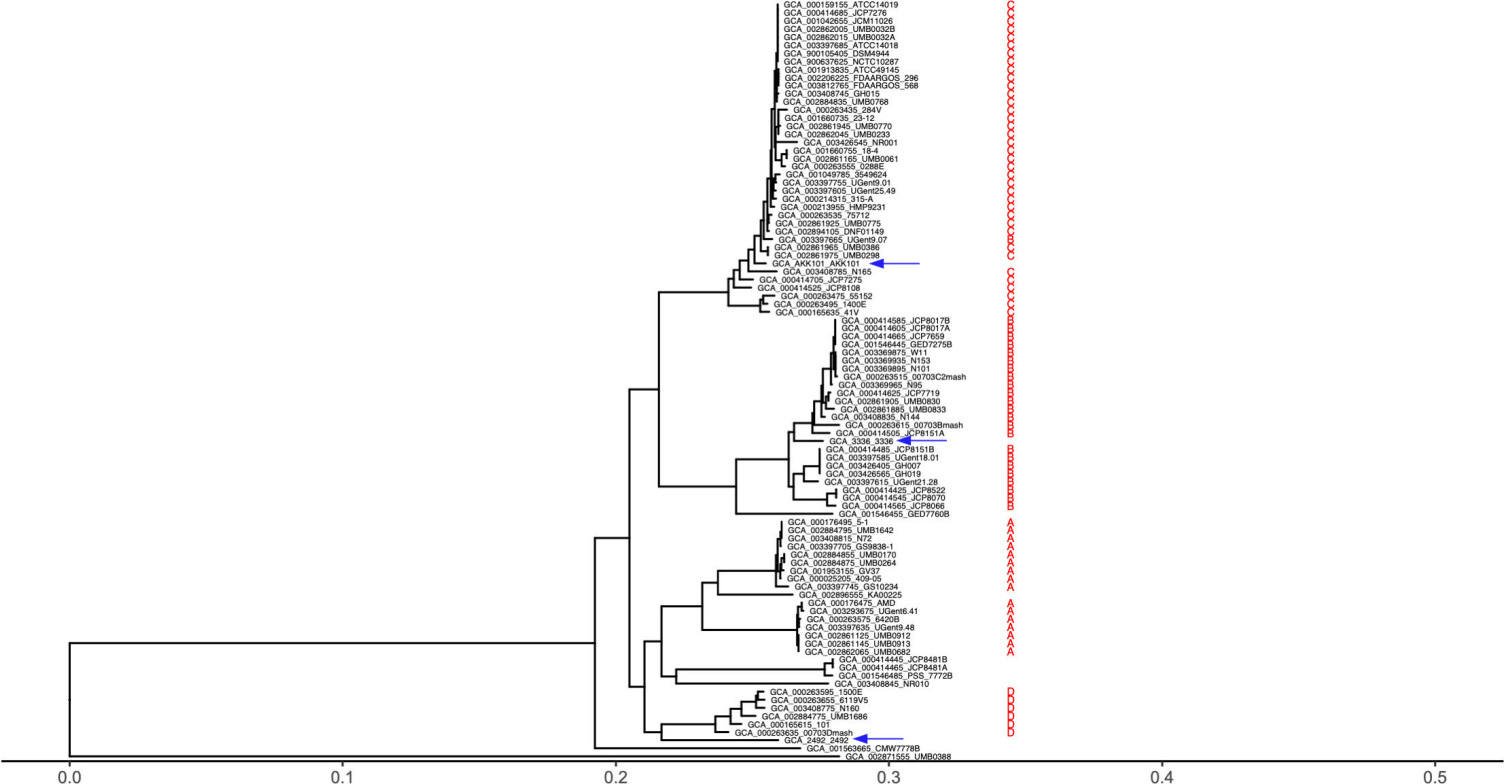
*cpn60* alignment to determine species designation of *Gardnerella* strains of interest. Neighbor-joining phylogenetic tree of full-length *cpn60* nucleotide sequences (1629 bp) of 91 *Gardnerella* spp. available genomes from GenBank. Clade distribution as described by (29). AKK101 most closely groups with clade C and strain 3336 with clade B. Strain 2492 is closest to clade D but may be outside of the original four clade designation. Regardless, each strain of interest is a distinct *Gardnerella* species. Arrows mark strains characterized in this study.

**Fig 2:**
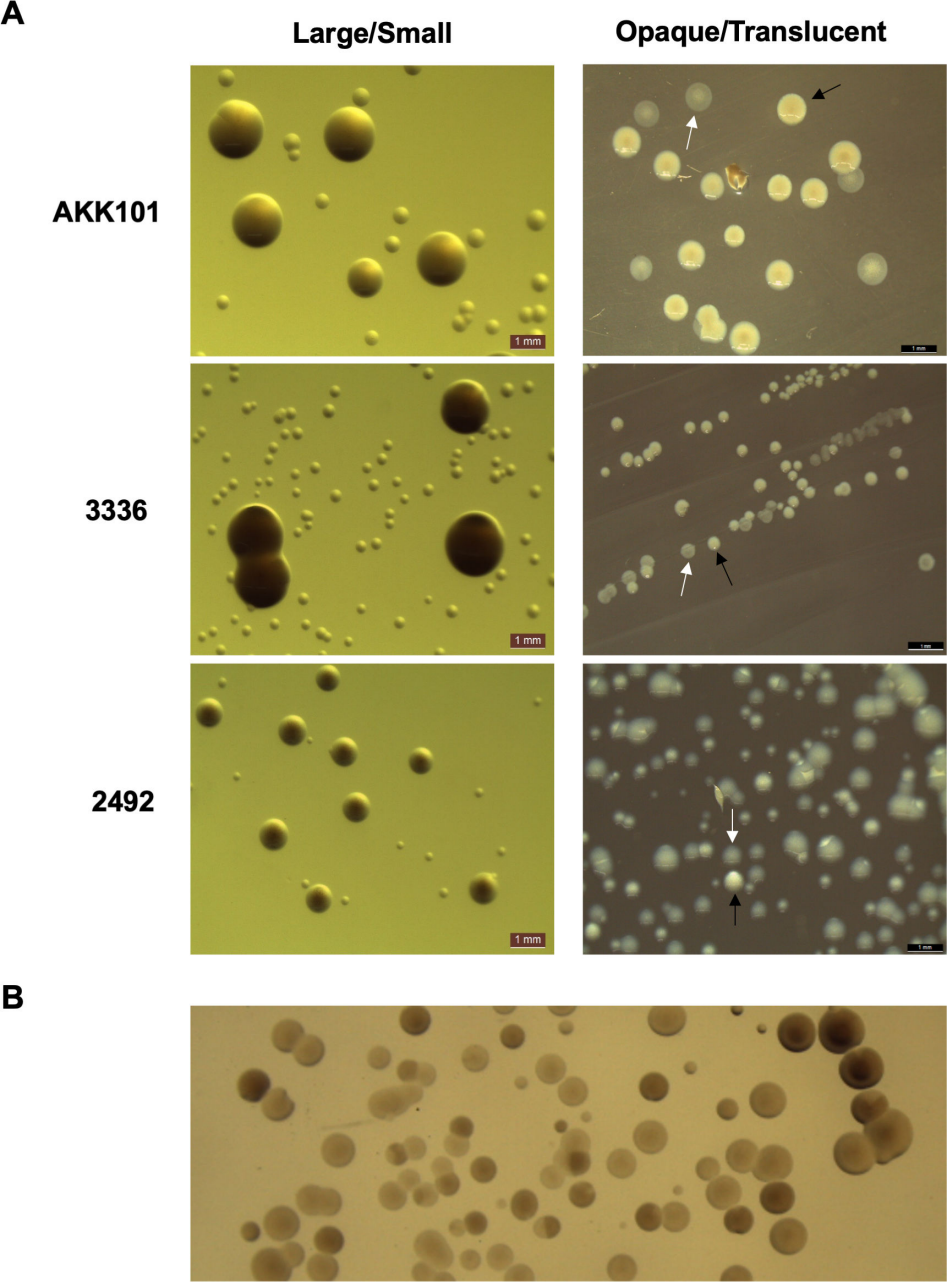
*Gardnerella spp*. exhibit variation in colony size and opacity. (**A**) (Left) Strains from three different *Gardnerella* species assembled into large (Lg) and small (Sm) colonies on BHIFF agar medium. (Right) Opaque (Op) and translucent (Tr) colonies were observed on sBHI plates. Black arrows indicate Op, and white arrows indicate Tr. (**B**) Colonies from strain AKK101 show a spectrum of opacity intensities. Images were taken with a Leica S8 APO stereo microscope.

Three *Gardnerella* strains classified as distinct species by *cpn60* alignment were chosen for detailed characterization (Fig. 1). During routine passage of strains on BHIFF agar, we observed the presence of colonies with distinct sizes. For each strain, we mixed large and small colony morphotypes in suspension and plated them on BHIFF agar to illustrate the relative sizes and appearance of the colonies (Fig. 2). The first morphotype, “large colony variant” (Lg), consisted of round, raised, colonies that were ~0.5–1.0 mm in diameter. The “small colony variant” (Sm) morphotype consisted of similarly shaped colonies that were significantly smaller in size, less than one-fourth the size of Lg colonies (Fig. 2A, left). On BHI_YDS_, sBHI, or HBT media, the difference in size between variants is not as notable. Sm colonies were either raised or flat. We also observed differences in colony opacity, most easily observed on sBHI medium (Fig. 2A, right). Opaque (Op) colonies were white to off-white, whereas translucent (Tr) colonies were gray to nearly clear. Opacity was observed as a spectrum of intensities rather than a strictly dichotomous phenotype (Fig. 2B). Although we found that large colonies were predominantly also opaque and small colonies were predominantly also translucent, we have observed large, translucent and small, opaque colonies (data not shown). This observation suggests that size and opacity phenotypes are independent of each other. We tested whether these variations were restricted to select strains or consistent across a variety of *Gardnerella* species and strains. In all 15 strains tested, including representatives from 10 different *Gardnerella* species, the variation of both colony size and opacity was readily apparent (Table S1).

**Fig 3:**
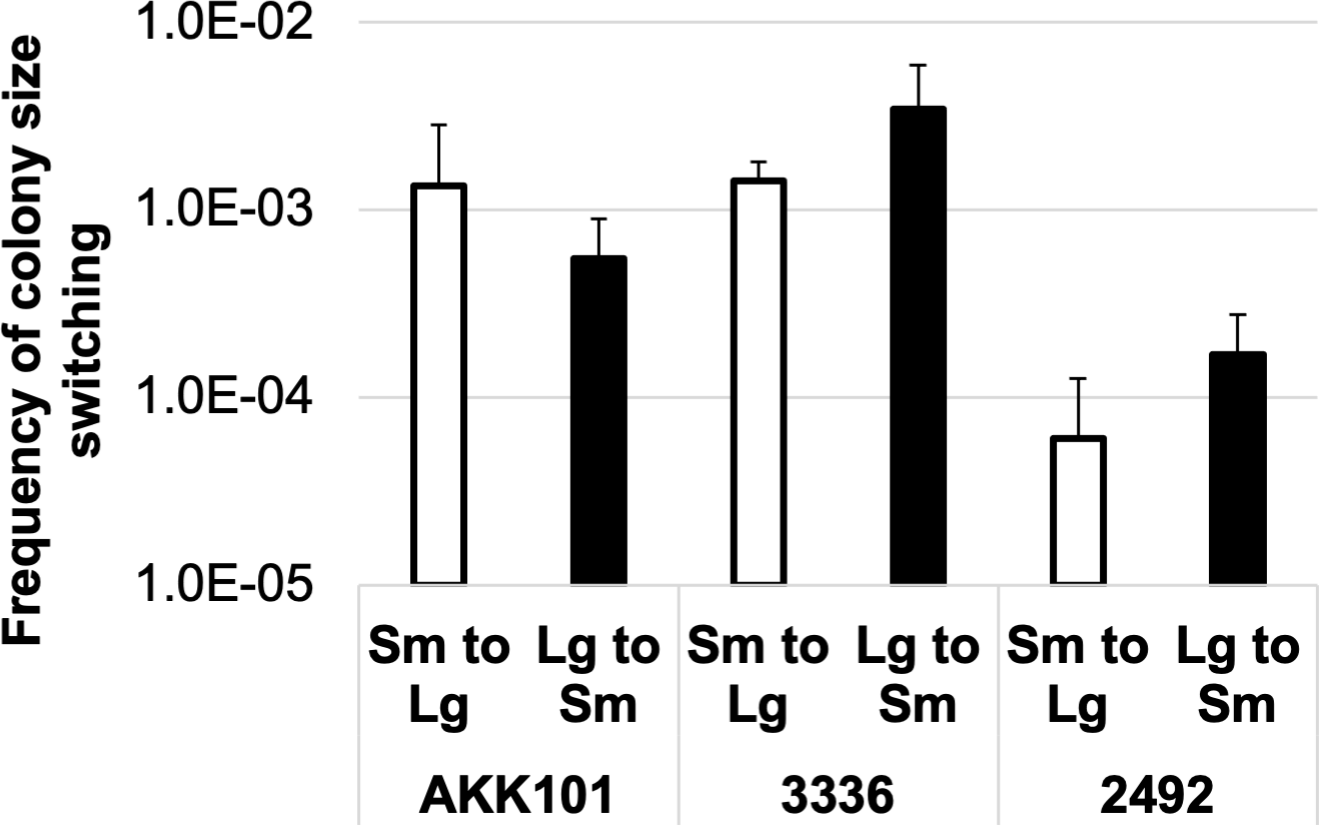
Phenotypic switching of colony size occurs at appreciable frequencies in both directions. Data are presented as an average of three independent experiments. Error bars represent the standard deviation between independent experiments.

For both size and opacity morphologies, we observed that the passage of single colonies consistently yielded a small fraction of the opposite variant (Lg gave rise to Sm, Tr gave rise to Op, etc), indicative of phase variation. To assess switch frequency, Lg and Sm phenotype colonies were incubated for 48 h on BHIFF and then replated. Quantification of switch frequencies is presented in Fig. 3. The switch frequency range was between 6 in 100,000 and 3.5 in 1,000, which is much higher than the rate of random mutation in bacteria (46). These data thus indicate that colony size phenotypes are under the control of high-frequency phase variation, which prompted us to test whether these variants were phenotypically distinct in other ways.

### Growth is faster for large colony variants than for small colony variants

**Fig 4:**
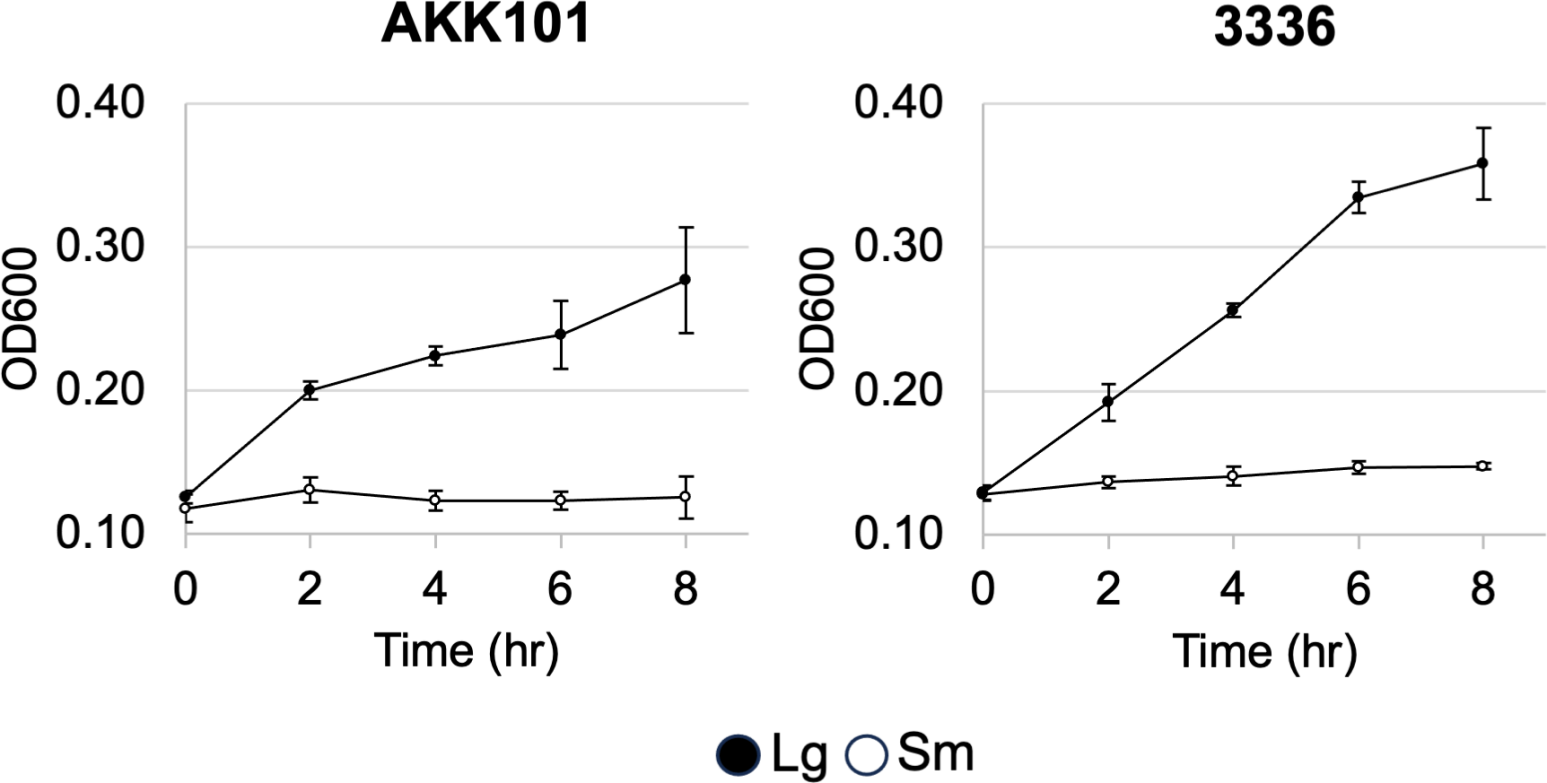
Large colony variants grow faster than small colony variants. Growth of each variant was quantified by measuring OD_600_ over a period of 8 h in BHIFF medium. Data are presented as an average of three independent experiments. Error bars represent the standard deviation across independent replicates.

The appreciable difference in colony size between variants on certain media suggested that the Sm variant was growing more slowly than the Lg colony variant. To quantify the difference in growth rate, we performed 8 h growth curves in BHIFF medium (Fig. 4). For both AKK101 and 3336, growth was faster for the Lg variant, with significant differences observed starting at 4 h (*P* < 0.0001 for both strains). These differences remained significant for the duration of the experiment. These data indicate that the differences in colony size for the variants reflect differences in the growth of the bacteria and not just differences in bacterial interactions during growth on agar.

### Small colony variants secrete greater amounts of vaginolysin than large variants

**Fig 5:**
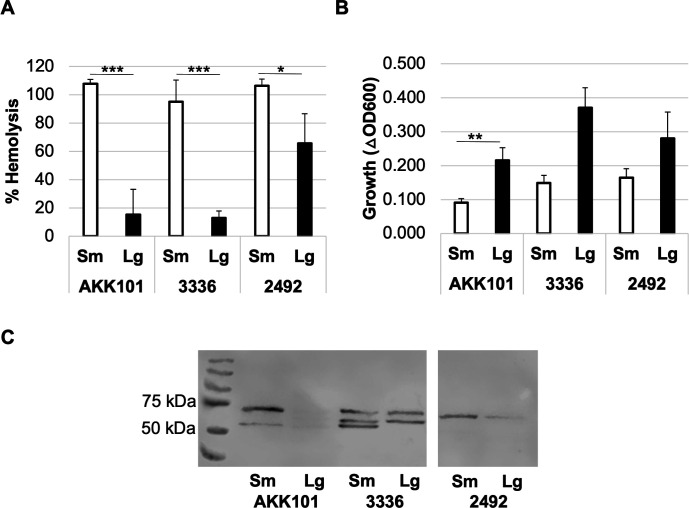
Supernatants from small variants elicit greater hemolysis. Bacteria were grown in BHI_YDS_ medium, and the culture supernatant was collected after 24 h. (**A**) Concentrated supernatant from triplicate wells was assayed for VLY production by percent hemolysis of human erythrocytes. (**B**) Strain growth was measured by the change in OD_600_ between inoculation and supernatant harvesting. Data from A and B are presented as an average of three independent experiments. Error bars represent standard deviation. *P < 0.05,**P < 0.01, ***P < 0.001. (**C**) Western blots of VLY in *Gardnerella* spp. supernatants. Reported VLY molecular weight is 57 kDa for the full-length protein (with signal sequence). It is unclear why multiple bands are present for some strains.

The small colony phenotype is associated with altered virulence factor production in some bacteria (47). Most *Gardnerella* strains produce the pore-forming toxin vaginolysin (VLY), which acts to lyse human epithelial cells, red blood cells, and other cell types. We sought to determine whether the secretion of vaginolysin differed between variants. Strains were grown in BHI_YDS_ medium, and the amount of VLY in culture supernatants at 24 h post-inoculation was assessed via hemolysis assays. The presence of VLY in culture supernatants was confirmed by western blotting (Fig. 5C; Fig. S1). In all strains tested, hemolysis was significantly greater (1.6-fold to 7.3-fold) in small colony variants (Fig. 5A), despite reduced growth relative to large colony variants (Fig. 5B). These data indicate that the phase variants differ not only in growth characteristics but also in virulence potential.

### Colony variants differ in their antagonism of vaginal commensals and pathogens

**Fig 6:**
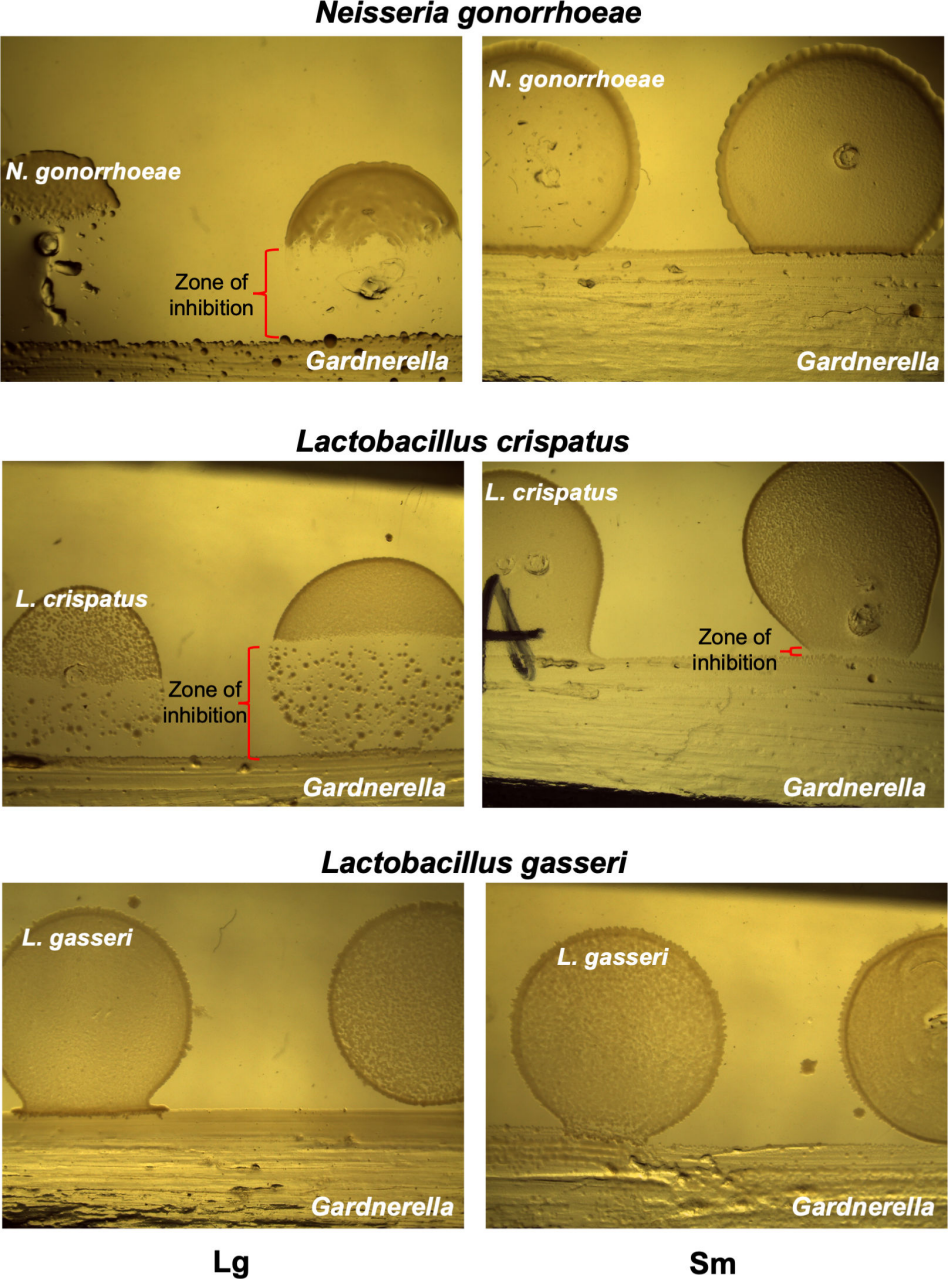
Colony variants differ in their antagonism of urogenital bacteria. *Gardnerella* colony variants from strain AKK101 were streaked onto BHI_YDS_ medium + 10% FBS (fetal; bovine serum) and grown for 24 h before adding a spot of diluted bacterial culture from *N. gonorrhoeae*, *L. crispatus*, or *L. gasseri* . After 24 h of co-culture, plates were assessed for a zone of clearing (red brackets) within the spot suggestive of growth inhibition. The results from a representative experiment are shown, but three independent experiments were performed.

Multispecies interactions are important determinants of vaginal health and disease, and the decline of *Lactobacillus* spp. is a key step in BV development (48, 49). We investigated the effects of *Gardnerella* variants from each species on the growth of representative commensal *Lactobacillus* species, *Lactobacillus crispatus* and *Lactobacillus gasseri*, and the urogenital pathogen, *N. gonorrhoeae*. These interactions are presented in Fig. 6 and Fig. S2. The *Gardnerella* variants were streaked on a BHI_YDS_ medium plate, and the following day, a *Lactobacillus* or *N. gonorrhoeae* culture was spotted adjacent to each streak. Inhibition was assessed by the presence of a zone of inhibition between the *Gardnerella* streak and the culture spot.

For *L. gasseri*, only the Sm variant of strain 3336 was inhibitory, albeit slightly. In contrast to this limited inhibition of *L. gasseri*, all of the strains characterized in this study exhibited some inhibition of *L. crispatus* growth, determined by the presence of a zone of inhibition with reduced *L. crispatus* density adjacent to the *Gardnerella* streak. For strains AKK101 and 3336, this inhibition was observed for both Lg and Sm variants. However, the zone of inhibition was significantly smaller for Sm variants. For strain 2492, inhibition was only observed for the Lg variant. The inhibition of *N. gonorrhoeae* by *Gardnerella* variants was substantially different from that of *L. crispatus*. The Lg variant of strains AKK101 and 3336 strongly inhibited *N. gonorrhoeae* growth, showing a large, complete (no single colonies) zone of inhibition. The Sm variant of these strains did not affect *N. gonorrhoeae* growth. Strain 2492 did not inhibit *N. gonorrhoeae*. These results demonstrate that *Gardnerella* spp. either have different responses to different bacteria or produce products that are inhibitory to select organisms. Furthermore, the production of the inhibitory substances varies between Lg and Sm colony phenotypes.

### Proteomic differences between Lg and sm variants

**Fig 7:**
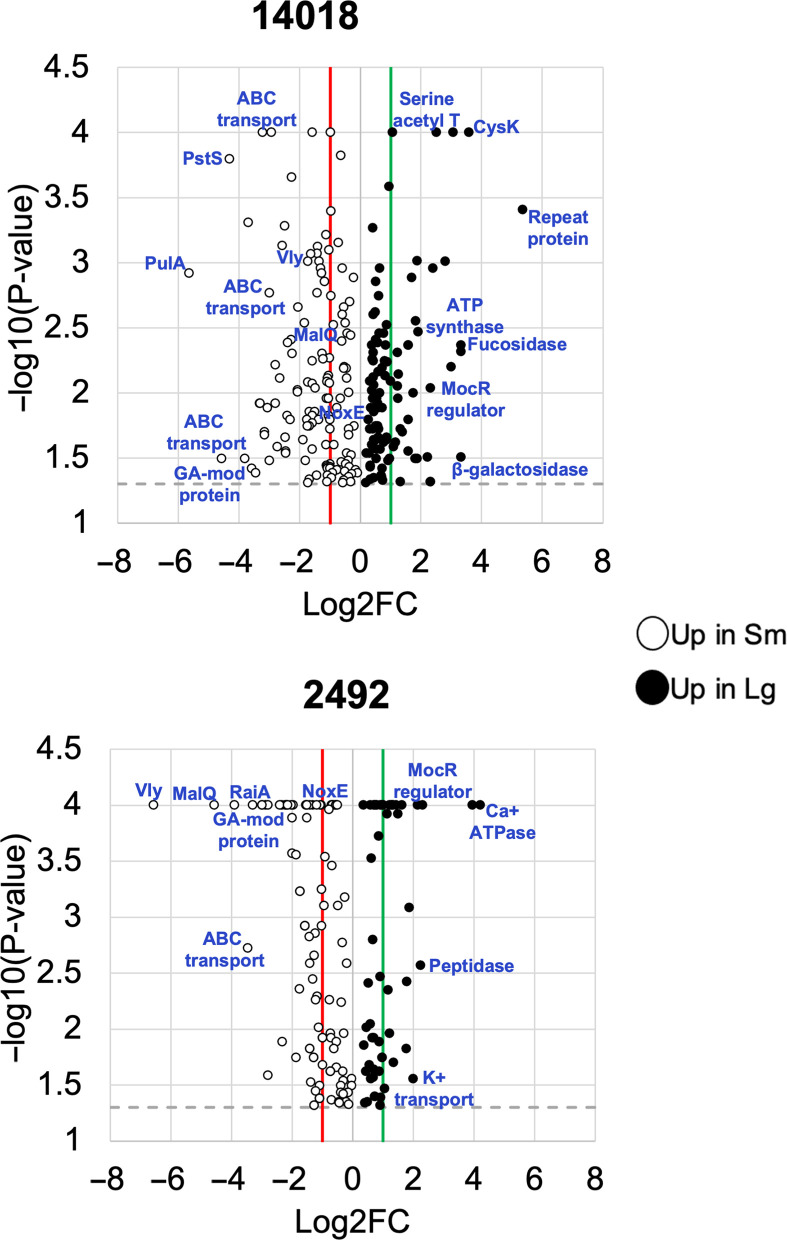
Colony variants differ in protein production. Volcano plots of all proteins differentially produced (*P* < 0.05) between colony variants in strains 14018 (top) and 2492 (bottom). Each circle represents one protein. Proteins accumulate in a linear fashion at a −log10(*P*-value) of 4 due to *P*-values lower than 0.00010 being assigned a value of 0.00010 (−log10(*P*-value) of 4). The gray-dashed line represents a *P*-value of 0.05; proteins above the line have a *P*-value less than 0.05. The red line represents a fold change of 2 for the Sm variant; proteins to the left of the line are increased in Sm by 2-fold or greater. The green line represents a fold change of 2 for the Lg variant; proteins to the right of the line are increased in Lg by 2-fold or greater.

To begin to dissect the factors contributing to the many observed phenotypic differences in colony variants, we performed a proteomics analysis on each variant from strains of two different species, 2492 and ATCC 14018. Strain ATCC 14018, which is classified as the same species as AKK101 (*Gardnerella vaginalis*), was chosen for inclusion, rather than the other strains presented in this study because a closed reference genome was available for this strain. All data presented are of proteins detected as significantly different (*P* < 0.05) between variants in analyses of three independent experiments (Fig. 7). The proteins with increased expression in Lg variants in both strains are presented in Table S2. These proteins are predominantly involved in amino acid and protein synthesis and protein folding (GroS, GroL). Interestingly, an aminotransferase, class I/II protein that is upregulated in both Lg variants may be a MocR family transcriptional regulator. The proteins with increased expression in Sm variants in both strains are presented in Table S3. Consistent with our data from Fig. 5, VLY expression was significantly increased in Sm variants relative to Lg, and this difference was greater in strain 2492 (95-fold) than in strain ATCC 14018 (3.3-fold). We also observed increased expression of proteins involved in glycogen breakdown (MalQ), phosphate transport (PstS, PhoU), oxygen tolerance (NoxE), nucleotide synthesis (HMPREF0421_20874, PyrH, NrdD, Gnd, PflB), cell wall synthesis (HMPREF0421_20394), DNA recombination and segregation (RecA, FtsK, HMPREF0421_20072), ABC (ATP-binding cassette) transport (HMPREF0421_20105, HMPREF0421_20232, HMPREF0421_20436, HMPREF0421_20240), and RNA posttranscriptional modification (HMPREF0421_20849). In addition to the similarly expressed proteins presented in Tables S2 and S3**,** each strain displayed unique differences in protein expression between Lg and Sm variants. The top 15 proteins with the highest Lg/Sm ratio are presented for strains 2492 and ATCC 14018 in Tables S4 and S5, respectively, and the top 15 proteins with the highest Sm/Lg ratio are presented in Tables S6 and S7. Lg variants in strain 2492 exhibited increased expression of a calcium-translocating ATPase (18.5-fold), a DNA topoisomerase (15.5-fold), ribonuclease J (4.8-fold), a C1-like peptidase (3.4-fold), and RpoA (2.4-fold). In strain ATCC 14018, Lg variants showed increased production of an internalin B repeat protein (43-fold), an alpha-L fucosidase (9.8-fold), ATP synthase domains (9.2 and 8.4-fold), and beta-galactosidase (4.7-fold). Of interest in strain 2492 Sm variants was the increased production of a ribosome hibernation promoting factor (9.9-fold), a predicted GA module surface protein (7-fold), and a universal stress family protein (4.7-fold). In Sm variants of ATCC 14018, expression levels of a glycogenase (50-fold), a GA module-containing protein (24-fold), and an arylsulfatase (10-fold) were significantly increased.

We next assigned all proteins with 1.5-fold or greater difference (*P* < 0.05) a KEGG (Kyoto Encyclopedia of Genes and Genomes) protein class to determine if there were any functional differences between variants (Fig. S3A). A greater proportion of proteins were assigned to the “genetic information processing” class in Lg (inner circles) compared with Sm variants (outer circles) for both strains. In Sm variants, the proportion of proteins left unclassified was greater than in Lg variants. Other functional differences observed were restricted to a single strain. For example, in ATCC 10418, the proportion of “energy metabolism” proteins was greater for the Lg variant while “transport” proteins were increased in the Sm variant. In 2492, “nucleotide metabolism” was increased in the Lg variant while “carbohydrate metabolism” was increased in the Sm variant. We also broke down the KEGG classes with the greatest number of protein assignments (transport, genetic information processing, amino acid metabolism, and carbohydrate metabolism) into subclasses to investigate whether there were any differences at this level (Fig. S3B). For the “transport” class, peptide and nickel transport proteins were only observed in Lg variants. For “genetic information processing,” proteins classified into DNA replication, DNA repair, ribosome biogenesis, and chromosome and associated proteins subclasses were increased in Sm variants while proteins assigned to tRNA biogenesis, translation, and chaperones and folding catalysts subclasses were increased in Lg variants. The “amino acid metabolism” subclasses of glycine, serine, threonine metabolism and lysine biosynthesis were increased in Sm variants. Finally, the glycolysis/gluconeogenesis subclass of “carbohydrate metabolism” was increased in Sm variants.

Collectively, these data demonstrate that Lg and Sm variants exhibit different metabolic profiles consistent with different growth characteristics and identify differentially expressed proteins likely to affect interactions with host cells. Furthermore, some differences between variants were found to be strain-specific and may reflect the different roles of *Gardnerella* species in host colonization and disease.

### Small colony variant survives longer than large variant in a human cervical explant infection model

**Fig 8:**
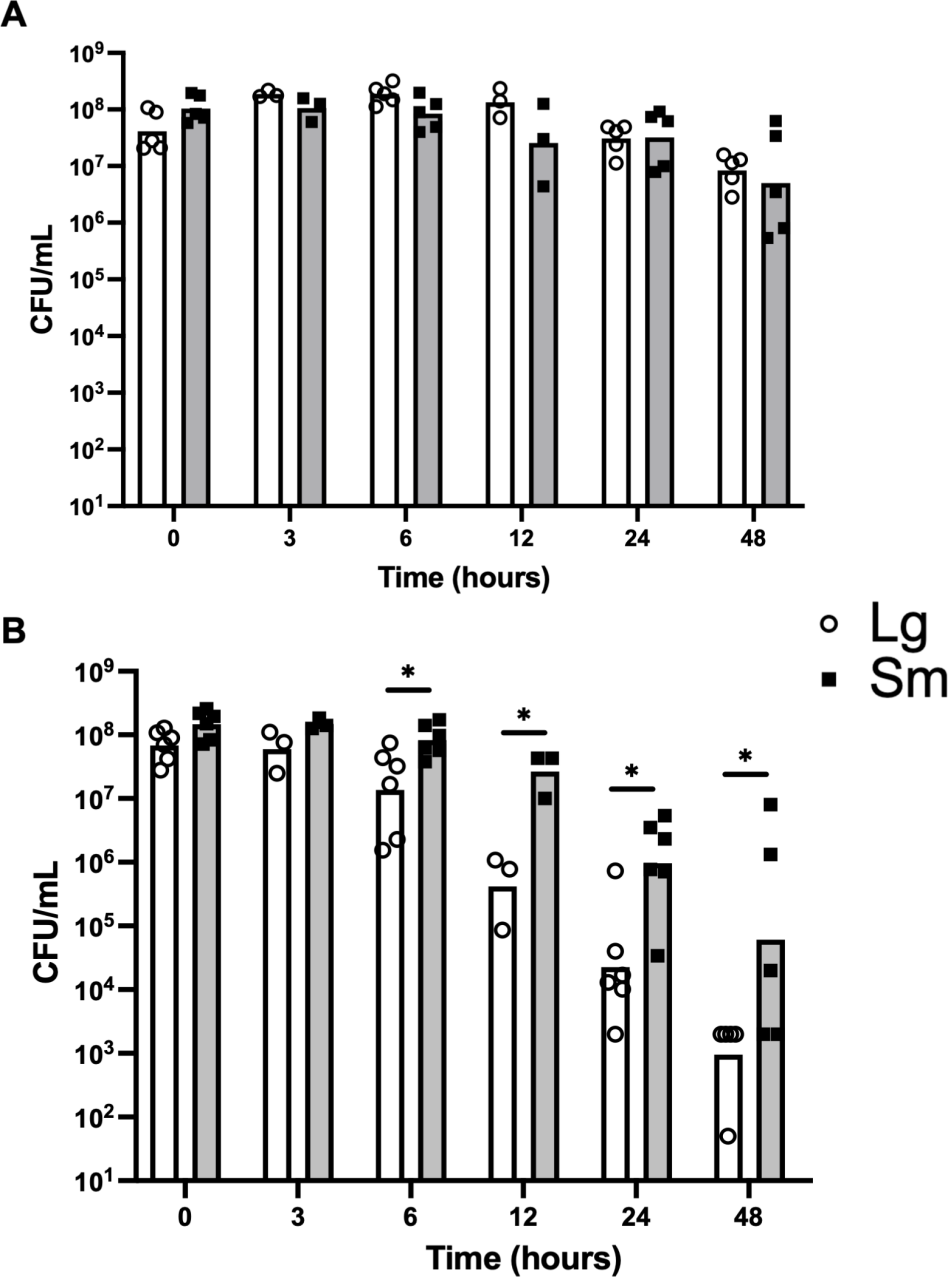
Small colony variants survive longer during experimental cervix infection. Survival over time of Lg and Sm variants from strain AKK101 in (**A**) CTCM (cervical tissue culture medium) alone or (**B**) in co-culture with human ectocervical tissue. Data are presented as geometric mean of at least three independent experiments. Independent experiments in tissue co-culture use tissue from different donors. *P < 0.05

The observation of multiple phenotypic differences led us to hypothesize that colony variants would interact differently with the human host. We tested this hypothesis using a human cervical explant infection model to compare the survival of colony variants. Biopsy punches of ectocervical tissue were infected with either Lg or Sm variants from strain AKK101, and the concentration of bacteria in the medium (CFU/mL) was quantified over time. In tissue medium alone (Fig. 8A), bacterial viability remained high up to 48 h post-infection, and there were no significant differences between colony variants. In contrast, during co-culture with ectocervical tissue, bacteria exhibited a notable decline in viability starting at 6 h post-infection (Fig. 8B). The decline was more severe for the Lg variant with a ~ 1 log difference relative to the Sm variant at 6 h (*P* = 0.022) and a ~ 2 log difference at 12, 24, and 48 h (*P* = 0.046, 0.016, and 0.046, respectively). These results suggest that the Sm variant of *Gardnerella* strain AKK101 is more resistant to host innate immunity.

### Variable homopolymer tracts are abundant in *Gardnerella* spp. genomes

**Fig 9:**
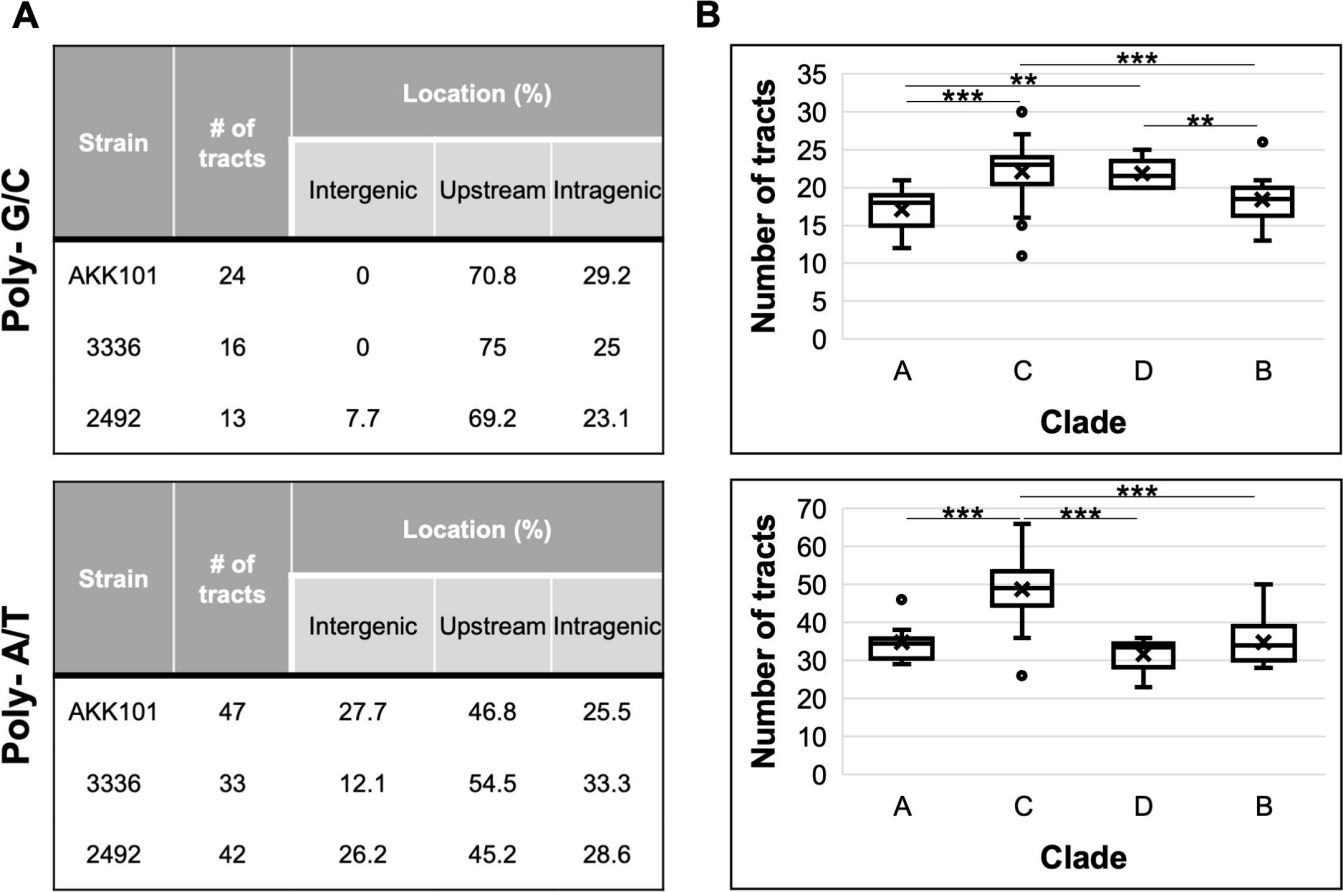
Homopolymer tract distribution in *Gardnerella* genomes. (**A**) Characteristics of homopolymer tracts in strains of interest. (**B**) Median tract number grouped by *Gardnerella cpn60* clade. The line within each box represents the median; the “X” represents the mean. Bars extending from boxes represent maximum and minimum values. Data points outside of bars are outliers. **P < 0.01, ***P < 0.001.

To define the genetic determinants of *Gardnerella* phase variation, we performed whole genome sequencing on variants from all strains of interest. Prior to genome comparisons between variants of the same strain for the detection of SNPs (single nucleotide polymorphisms) and insertions/deletions, we noted and characterized the ample distribution of long homopolymeric tracts (≥ 8 base pairs) throughout all analyzed genomes (Fig. 9A). In each strain, the majority of tracts (~70% for G/C and ~50% for A/T) were localized to elements upstream (<300 base pairs from the start site) of a predicted open reading frame (ORF). Approximately one-quarter of tracts were located within a predicted ORF. Many tracts were associated with ORFs annotated as hypothetical proteins, but these proteins commonly contained motifs associated with cell surface proteins including predicted Rib domains, GA modules, FIVAR domains, InlB B-repeat regions, and LPXTG sortase cleavage sites. Other ORFs associated with homopolymer tracts included genes predicted to function as methyltransferases, peptidases, transporters, transcriptional regulators, pullulanases, ribosomal proteins, N-acetyltransferases, and toxin-antitoxin proteins.

Further analysis of the number of homopolymeric tracts in publicly available *Gardnerella* genomes (*n* = 91) on NCBI (National Center for Biotechnology Information) showed median tract counts of 20 and 38 for G/C and A/T tracts, respectively. That there were higher numbers of A/T tracts in *Gardnerella* genomes relative to G/C tracts is consistent with reports that A/T homopolymers are relatively abundant in prokaryotic genomes (50). The number of long homopolymers in *Gardnerella* is comparable with what is observed in *N. gonorrhoeae* strains, where 29 genes were found to vary by changes to the number of repeats within homopolymeric tracts (51). These results indicate that an abundance of genomic homopolymer tracts is a conserved attribute of the *Gardnerella* genus. However, differences in tract abundance were observed when the strains were broken up by clade (Fig. 9B). Clade C, which includes *G. vaginalis* and species group 2, had a significantly higher A/T tract median (49) compared with the three other clades (33.5–34.5). With respect to G/C tracts, clades C and D had higher median tract counts (21.5–23) relative to clades A and B (18–18.5).

Although several homopolymer tracts were called as variable between colony variants in our whole genome sequencing analyses, next-generation sequencing platforms can be unreliable in detecting differences in these low-complexity genetic elements (52, 53). To verify that homopolymer tracts ≥ 8 base pairs are variable in *Gardnerella*, we selected a tract for Sanger sequencing. The chosen tract was associated with a predicted GA module containing protein (GAVG_0299 in AP012332). A single colony was re-streaked onto an agar plate, and several colonies of the Lg phenotype were picked for sequencing of the region containing the homopolymer tract. Colony sequencing was performed on strain 14018, one of the strains used for proteomics analysis, as well as on strain AKK101. In strain 14018, nine colonies had a tract length of 9 base pairs and one colony had a tract length of 10 base pairs. In strain AKK101, seven colonies had a tract length of 9 base pairs and two colonies had a tract length of 10 base pairs.

These results demonstrate that homopolymer tracts are abundant and variable in *Gardnerella* genomes. Due to the association of homopolymer tracts with slipped strand mispairing and phase/antigenic variation, these findings highlight previously unrecognized clonal heterogeneity occurring in *Gardnerella* isolates.

### Whole genome sequencing identifies multiple genetic changes in *Gardnerella* colony variants

We questioned whether SNPs and insertions/deletions not associated with a homopolymer tract could be contributing to the colony size phenotype. We were particularly interested in mutations that were present in all analyzed strains. There were 17, 23, and 18 SNPs called between colony variants in strains AKK101, 3336, and 2492, respectively. Insertions/deletions totaled 4, 2, and 8 in the same strains. Among these mutations, only one gene was commonly mutated across all three strains. This gene is annotated as *ulaA*, an ascorbate-specific PTS transporter (GAVG_0722 in AP012332). In strain AKK101, the Sm colony variant had a single nucleotide insertion 869 base pairs into the ORF, resulting in a predicted premature stop codon at 879 base pairs. In strain 3336, a SNP at position 875 in the ORF resulted in a missense mutation giving rise to a cysteine in the Lg variant and a phenylalanine in the Sm variant. In strain 2492, a missense mutation at position 876 generated a lysine codon in the Sm variant and a phenylalanine in the Lg variant.

To test whether *ulaA* mutation may be causal in colony size phenotype, a single Sm colony from strain AKK101 was passaged onto agar, and the region of *ulaA* containing the detected mutation was sequenced in the parent Sm colony and the daughter colonies (three each) of either the Sm or Lg phenotype. We reasoned that the daughter Lg colony would show differences in the *ulaA* sequence when compared with the parent or daughter Sm colonies. However, the sequenced region was the same for all colonies. We conclude, therefore, that *ulaA* mutation is not causal in colony size phenotype for strain AKK101.

In sum, the data suggest that colony variation in *Gardnerella* spp. is a complex phenomenon, making identification of causal genetic factors difficult. Given the multiple degrees of opacity we have observed and the differences in colony color, colony opacity is likely a polygenic phenotype. Furthermore, our data indicate that there is ample phase variation occurring that we are unable to observe at the colony level.

## DISCUSSION

The mechanisms used by *Gardnerella* in establishing infection and promoting the dysbiosis of BV are just beginning to be understood. Nearly all women are colonized with *Gardnerella* spp., but approximately one-third of women of reproductive age develop BV (26). In the days preceding symptomatic BV, the number of *Gardnerella* cells greatly increases and the number of *Lactobacillus* cells plummets (10). Vaginal pH increases due to the loss of lactic acid production. *Gardnerella* build a biofilm that incorporates various anaerobic bacteria, and there is evidence of crossfeeding with *Gardnerella* releasing amino acids used by the other species (54, 55). Some *Gardnerella* species produce sialidase that breaks down cervical mucus (17). *Gardnerella* are also known to break down glycogen, and these various metabolic activities of *Gardnerella* likely affect the growth of other BV-associated bacteria (15). Furthermore, patients with BV are more likely to acquire sexually transmitted infections including gonorrhea and chlamydia, and the mechanisms involved have been proposed to involve metabolic interactions or reductions in immune cell presence and function affected by *Gardnerella* factors (56, 57). The role of the *Gardnerella* toxin vaginolysin in infections is not clear. In this context, our identification of *Gardnerella* phase variants that are affected in the inhibition of *Lactobacillus* spp. and *N. gonorrhoeae*, vaginolysin production, predicted adhesin production, physiology, and survival in cervical tissue, is an important step in understanding the *Gardnerella* infection process.

We identified both Lg and Sm colony size variation and Op and Tr colony opacity variation. Colony variability in *Gardnerella* was previously noted by Robinson et al. but not further characterized (14). In other bacterial species, colony opacity has been found to be due to bacterium-bacterium interactions or the production of polysaccharides or other biofilm-related molecules (58–61). We have not yet identified the factors that affect *Gardnerella* colony opacity. Lg and Sm variants were found to differ in metabolic proteins, growth rate, toxin production, and cervix infection *ex vivo*. We measured the colony variant switch frequencies and found that the rates were on the order of 10^−5^ to 10^−3^, switching from Sm to Lg or Lg to Sm in a 48-h period. This rate is higher than would be expected for random mutation, suggesting that a specialized mechanism involving repeat sequences or recombination methods may be involved (62). Our proteomic and genomic analyses did not reveal an obvious switch mechanism. However, for strain 2492, the proteomics data identified possible mechanisms for reduced growth. The Sm variant showed increased production of a ribosome hibernation factor and a universal stress protein, both associated with decreased metabolism (63, 64). However, it is not clear how levels of these factors are regulated, and they were not identified in the ATCC 14018 proteomic analysis, suggesting that different factors may be utilized in different species.

In our characterization of colony size morphotypes, we observed that Lg colony variants displayed a faster growth rate and diminished cytotoxin production relative to Sm variants (Fig. 4 and 5). The ability to inhibit the growth of other bacterial species varied between morphotypes and strains (Fig. 6; Fig. S2). The proteomic profiles of variants were also distinct (Fig. 7; Tables S2 and S3). Although proteins involved in amino acid and protein synthesis were increased most (highest fold change) in Lg colonies, Sm colonies of both strains showed a more varied profile with increased representation of proteins related to glycogen breakdown, phosphate transport, oxygen tolerance, nucleotide synthesis, cell wall synthesis, DNA recombination, and RNA posttranscriptional modification. When all significantly increased proteins were grouped into functional categories, the proportion of proteins associated with genetic information processing was increased in Lg variants (Fig. S3). Importantly, a majority of the proteomic differences between variants were strain- and thus species-specific (Tables S4 to S7). These data suggest two things. First, as has been observed by others, *Gardnerella* species are profoundly phenotypically distinct (65). Second, colony size variants exhibit distinct physiologies, suggesting each may be better suited to survive under different environmental conditions, and differentially produce factors that would likely impact their interactions within the host.

**Fig 10:**
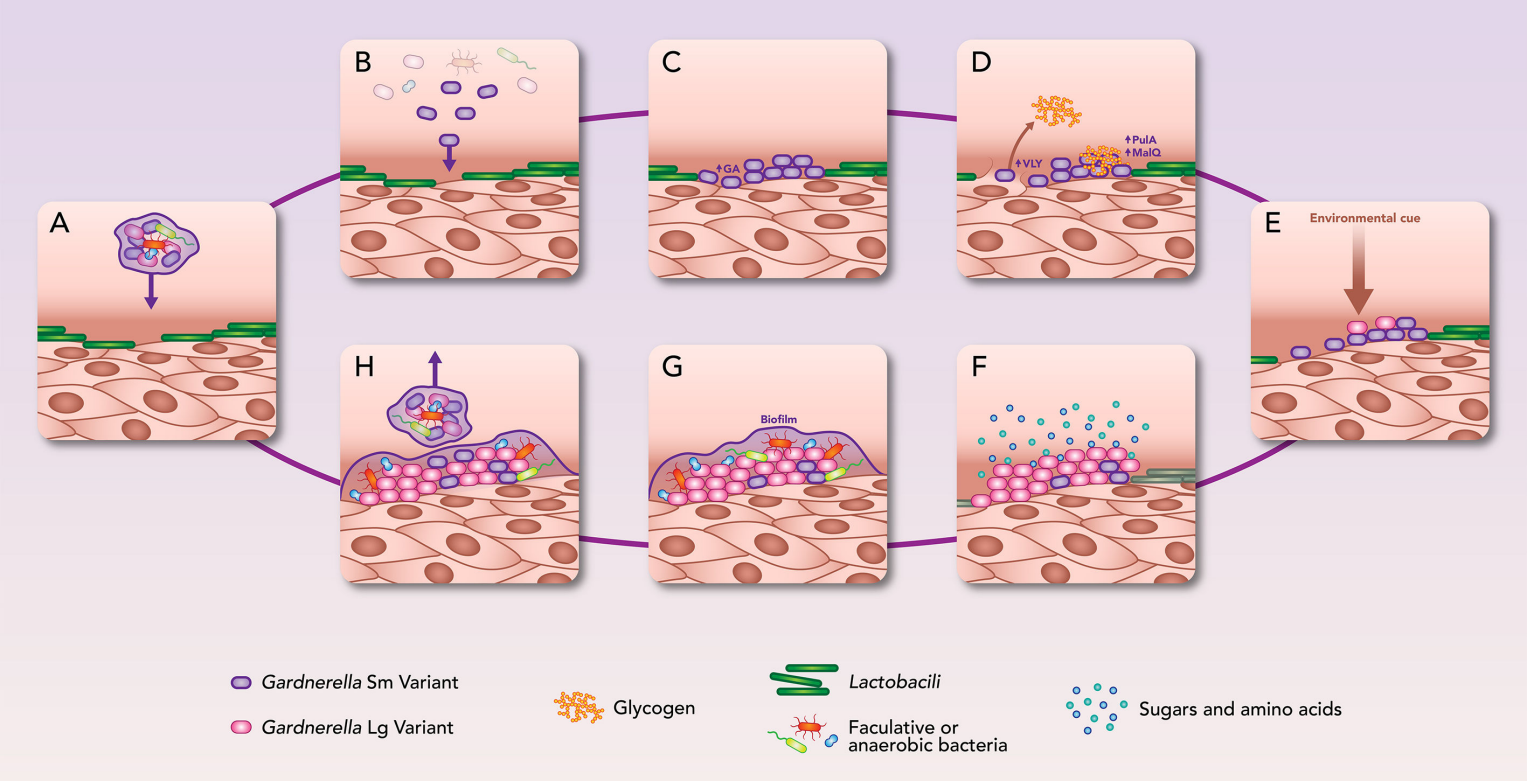
*Gardnerella* colony variant infection cycle hypothesis. (**A**) The BV biofilm encasing both Lg and Sm *Gardnerella* variants and other BV-associated bacteria enters the vaginal canal. (**B**) Upon biofilm dispersal, the Sm variant withstands the host innate immune defenses. (**C**) Increased expression of GA-module-containing proteins enhances autoaggregation and subsequent Sm adhesion to the host epithelium. (**D**) Secretion of vaginolysin toxin releases glycogen stored in epithelial cells, which Sm uses as a nutrient source through increased PulA and MalQ production. (**E**) An environmental cue triggers phenotype switching from the Sm to Lg variant. (**F**) The Lg variant produces sugars and amino acids that support the growth of *Prevotella* and other anaerobes. The presence of Lg is inhibitory toward Lactobacilli. (**G**) Lg initiates biofilm production and other BV-associated bacteria become incorporated. (**H**) Maturation of the BV biofilm gives rise to Sm variants. Biofilm aggregates, either themselves or attached to shed epithelial cells (clue cells), are transmitted to the next host.

The distribution of *Gardnerella* spp. colony phenotypes *in vivo*, and thus their role(s) in vaginal health and disease, is currently unknown. However, the observed differences between Lg and Sm colony variants suggest two possibilities. First, it is possible that these different forms of the bacteria represent different stages in an infection cycle (Fig. 10). Perhaps *Gardnerella* Sm variants are similar to *Chlamydia trachomatis* elementary bodies, the infectious but non-growing form of the bacteria (66). In this scenario, the Sm variant would be the initiator of infection. The increased production of the GA-module proteins may promote adhesion to host cells. Through slower growth kinetics and increased VLY production, the Sm variant may be more resistant to the host innate immune response. Its enhanced production of glycogenases could promote the breakdown of glycogen into substrates able to be used by the Lg variant and potentially other BV-associated bacteria. In contrast, the faster-growing Lg variant may be able to outcompete vaginal competitors for nutrient sources, inhibit lactobacilli, form the biofilm mass that supports other BV-associated species, and support transmission via aggregates in vaginal secretions (67). Alternatively, it is possible that Sm variants represent a persistent form of the bacteria. BV patients show a high rate of relapse within a few months following antibiotic treatment (68). The slow-growing Sm variants may be less likely to be eliminated by antibiotic treatment and may be responsible for disease recurrence. Sm variants could also occupy a distinct niche or produce fewer immunostimulatory molecules and thereby avoid immune clearance. Our finding that the Sm variants show increased survival in primary human cervical tissue is consistent with Sm variants acting as persisters (Fig. 8).

Whole genome comparisons revealed an abundance of homopolymeric tracts within *Gardnerella* spp. genomes and provided evidence of their variation. Homopolymeric tracts longer than 8 bp are likely to undergo gain or loss of nucleotides by slipped-strand mispairing during chromosome replication, which may result in increased or decreased gene expression or gain or loss of protein translation due to frameshift mutations (38). A number of the genes carrying polymeric tracts appear to be surface proteins and possible adhesins. We used Sanger sequencing to demonstrate that the repeat in one of these genes, encoding a GA-module protein, undergoes length variation *in vitro*. The identification of these variable genes provides targets to study possible surface antigens that may be recognized by the host immune system and surface proteins that may act in interactions with host cells. With both colony variants and homopolymeric repeat variants, *Gardnerella* colonies will contain multiple phenotypically distinct types. Likewise, biofilm aggregates in infection may contain multiple variants transmitted between individuals.

In sum, the data in this manuscript present a novel account of *Gardnerella* heterogeneity. As suggested previously, Sm colonies may represent a developmental stage for the bacteria, facilitating particular steps in the infection process. If true, this would be a paradigm shift in our understanding of how *Gardnerella* contribute to the development of BV. The existence of a slower-growing small colony variant may also explain the high rate of BV recurrence, as Sm colonies in other bacteria are associated with persistence and chronic disease states (69). Quantifying the predominance of each variant *in vivo*, at various points during incident and recurrent BV, and testing whether certain environmental factors alter the predominance of each variant will be important next steps for hypothesis testing. Furthermore, phase variation of surface proteins *in vivo* could facilitate evasion of immune responses during BV, promoting persistence and/or recurrence/re-infection. Identifying the repertoire of surface proteins susceptible to phase variation in each species will be important for the rational design of *Gardnerella*-targeting BV therapeutics.

## MATERIALS AND METHODS

### Strains and culture conditions

*Gardnerella* spp. strains 3336 and 2492 were isolated from women attending the Seattle Sexually Transmitted Disease Clinic between 1978 and 1984. Strain AKK101 is a spontaneous erythromycin-resistant derivative of strain Gv1, which is described in Bohr et al 2020. The sources of these strains and others referenced in the manuscript are outlined in the acknowledgments section. Various media were used for *Gardnerella* growth and variant distinction. These include HBT (human blood Tween) bilayer medium, BHIFF (brain heart infusion (BHI) broth with 10% FBS and 5% Fildes Enrichment), sBHI (BHI with 1.0% [wt/vol] gelatin, 1.0% yeast extract, 0.1% starch, and 0.1% dextrose) broth or agar supplemented with 10% FBS, and BHI_YDS_ (BHI with 0.5% yeast extract, 0.2% dextrose, 0.1% starch) broth or agar supplemented with 10% FBS. The culture conditions used for a given experiment are detailed in each respective section below.

*N. gonorrhoeae* strain MS11 was routinely cultured on GCB (gonococcal base) agar (Difco Laboratories) with Kellogg’s supplements I & II and incubated at 37°C with 5% CO_2_(70). *L. crispatus* 33820 (acquired from ATCC) and *L. gasseri* 33323 were cultured on MRS (De Man–Rogosa–Sharpe) agar at 37°C with 5% CO_2_.

### Clade and species determination

*Gardnerella* genomes (*n* = 91) were downloaded from NCBI and were annotated using Prokka (71). Sequences from genomes that were annotated as “60 kDa chaperonin 1” by Prokka were used for constructing the phylogenetic tree. Phylogeny.fr was used to generate the phylogenetic tree, and ggtree was used to visualize and annotate the tree (72, 73). Clade and species designations were assigned manually based on the strategies used in (19) (clades 1–4) (29), (clades A-D), and (25) (13 species designations, four named) (19, 25, 29).

### Growth rate determination

Colony variants were steaked from frozen stocks onto HBT plates and incubated at 37°C with 5% CO_2_ overnight (~16 h). Bacteria were then collected into BHIFF medium and adjusted to an initial OD_600_ of 0.12 in 4 mL of medium. Bacteria were incubated statically at 37°C with 5% CO2 for 8 h. Cultures were vortexed every 2 h, and 100 µL was removed to measure the absorbance at OD_600_ using a BioTek Synergy HT plate reader.

### Switch frequency

Freezer stocks of colony variants were steaked onto HBT plates and incubated at 37°C with 5% CO_2_ for ~24 h. To ensure collection of the desired phenotype, growth from HBT was swabbed into BHI broth, serially diluted, and plated onto BHIFF agar, where colony size phenotypes are best differentiated. BHIFF plates were incubated at 37°C with 5% CO_2_ for ~48 h, and then, the colony phenotype was assessed using a Leica S8 APO stereo microscope. Colonies (10–60) of each phenotype were collected on sterile filter paper and then added to BHI broth for serial dilution and plating onto BHIFF. Plates were incubated at 37°C with 5% CO_2_ for ~48 h, and the number of colonies of each phenotype was counted. Values for switch frequency represent the fraction of colonies that switched from the originally collected phenotype to the opposite phenotype.

### VLY hemolysis assay

*Gardnerella* strains were streaked onto HBT plates from freezer stocks and grown overnight at 37°C with 5% CO_2_. Growth was swabbed into BHI_YDS_ medium, and suspensions were adjusted to an OD_600_ of 0.1. Replicates of 200 µL of inoculum were placed in the wells of a 96-well tissue culture-treated plate and grown stationary at 37°C with 5% CO_2_ for 21–24 h. The initial and final ODs were measured, and the culture supernatant from triplicate wells was combined. Cells were pelleted and 500 µl of supernatant was concentrated with a 30 kD cut-off column (Amicon Ultra 0.5 mL). Samples were stored at −80°C prior to analysis. Blood was obtained from healthy human donors according to a protocol approved by the University of Wisconsin-Madison Institutional Review Board. Erythrocytes were collected by centrifugation (500 × *g* for 5 min), plasma was removed, and the cells were washed three times with sterile PBS. A 1% solution of erythrocytes was prepared. Concentrated supernatant was serially diluted with PBS, and 100 µL of each dilution was mixed with 100 µL of the 1% erythrocyte suspension in a V-bottomed 96-well plate. Positive and negative controls for hemolysis were a 0.1% solution of Triton X-100 (Tx) and PBS, respectively. The plate was incubated for 30 min at 37°C with 5% CO_2_ and then centrifuged for 10 min at 2,000 rpm to pellet intact erythrocytes. Supernatant was transferred to a flat-bottomed 96-well plate, and the absorbance (A) at OD_415_ was measured. Percent hemolysis was calculated as (A_sample_ – A_PBS_)/ (A_Tx_ – A_PBS_) × 100. Data presented in Fig. 5A are from the same dilution.

### VLY western blot

*Gardnerella* supernatants were prepared as outlined in the hemolysis section. Concentrated supernatants were mixed with SDS-loading dye and boiled for 5 min. Equivalent volumes of samples were run on 10% SDS-polyacrylamide gels, transferred to PVDF membranes (BioRad), and western blots were performed essentially as described previously (74). Primary VLY antibody 23A2 (Absolute Antibody) was used at a 1:10,000 dilution, and secondary goat anti-mouse HRP antibody (Santa Cruz Biotechnology) was used at a 1:20,000 dilution (75). Millipore chemiluminescent HRP substrate was used per the manufacturer’s instructions. Membranes were imaged with the Licor Odyssey Fc System.

### Bacterial antagonism

Freezer stocks of small and large colony variants of *Gardnerella* were streaked onto HBT plates and incubated at 37°C with 5% CO_2_ for ~24 h. Growth was swabbed from plates into BHI broth and then streaked onto BHI_YDS_ agar with 10% FBS. Plates were incubated at 37°C with 5% CO_2_ for ~24 h. After 24 h of growth, a 5 μL spot of serially diluted *N. gonorrhoeae*, *L. crispatus*, or *L. gasseri* was added perpendicular to the *Gardnerella* streak. Dilutions of *N. gonorrhoeae and L. gasseri* were prepared from 24-h growth on agar plates swabbed into BHI broth. *L. crispatus*, which did not consistently grow on MRS plates, was prepared from freezer stocks. Bacteria from the streak (*Gardnerella*) and the spot (*N. gonorrhoeae*, *L. crispatus*, or *L. gasseri*) were co-incubated on the BHI_YDS_ plate at 37°C with 5% CO_2_ for ~24 h before imaging on a Leica S8 APO stereo microscope to assess for a zone of clearing within the spot, indicative of *Gardnerella*-mediated growth inhibition.

### Preparation of whole cell lysates for proteomics analysis

Large and small colony variants of *Gardnerella* strains ATCC 14018 and 2492 were streaked onto HBT plates from freezer stocks and grown overnight at 37°C with 5% CO_2_. They were re-streaked onto BHI_YDS_ plates containing 10% FBS and incubated for 1–2 days. The growth was swabbed into cold sterile PBS, and the cells were pelleted at 4°C. Supernatant was removed, and the cells were washed three times in cold PBS with final resuspension in 50 mM sodium phosphate buffer (pH 7.5) with a 1× Roche protease inhibitor cocktail (2492) or a Pierce protease inhibitor tablet (ATCC 14018). The cells were lysed by sonication with a Branson digital sonicator (30 sec at 40% amplitude with a 1 sec on/off pulse), and insoluble material was removed by centrifugation (10 min at 13,000 rpm). Supernatant was transferred to a fresh tube, and protein concentration was determined by Bradford assay. Samples were stored at −20°C prior to analysis. Three biological replicates were prepared for each strain and variant.

### Proteomics analysis

#### Enzymatic “in liquid” digestion

Protein aliquots (50 µg) were incubated for 60 minutes on ice in 10% TCA and 33% (2492) or 50% (ATCC 14018) acetone (final vol:vol) to precipitate proteins, spun for 10 min at room temperature with max speed (16,000 × *g*), and then pellets were washed twice with cold acetone. Protein extracts were re-solubilized and denatured in 15 (2492) or 20 (ATCC 14018) μL of 8 M urea in 50 mM NH_4_HCO_3_ (pH 8.5) and then diluted to 60 µL for the reduction step. This dilution was in 2.5 µL of 25 mM DTT and 42.5 µL of 25 mM NH_4_HCO_3_, pH 8.5 for 2492 and 2.5 µL of 25 mM DTT, 5 µL of methanol, and 32.5 µL of 25 mM NH_4_HCO_3_, pH 8.5, for ATCC 14018. Diluted extracts were incubated at 56°C for 15 min and cooled on ice to room temperature. Iodoacetaminde (3 µL of 55 mM) was added for alkylation and incubated in the dark at room temperature for 15 min. The reaction was quenched by adding 8 µL of 25 mM DTT. Finally, 8 µL of Trypsin/LysC solution (100 ng/µL 1:1 Trypsin (Promega) and LysC (FujiFilm) mixed in 25 mM NH_4_HCO_3_) and 21 µL of 25 mM NH_4_HCO_3_ (pH 8.5) was added to 100 µL final volume. Digestion proceeded for 2 h at 42°C; then, an additional 4 µL of trypsin/LysC mix was added, and digestion continued overnight at 37°C. The reaction was terminated by acidification with 2.5% trifluoroacetic acid (TFA, 0.3% final concentration).

#### NanoLC-MS/MS

Digests were desalted using Agilent Bond Elute OMIX C18 SPE pipette tips per manufacturer protocol, eluted in 10 µL of 60%/40%/0.1% ACN/H_2_O/TFA, and dried to completion in a speed-vac. Finally, samples were reconstituted in 40 µL of 0.1% formic acid. Peptides were analyzed by nanoLC-MS/MS using the Agilent 1100 nanoflow system (Agilent) connected to a hybrid linear ion trap-orbitrap mass spectrometer (LTQ-Orbitrap Elite, Thermo Fisher Scientific) equipped with an EASY-Spray electrospray source held at constant 35°C. Chromatography of peptides prior to mass spectral analysis was accomplished using a capillary emitter column (PepMap C18, 3 µM, 100 Å, 150 × 0.075 mm, Thermo Fisher Scientific) onto which 2 µL of extracted peptides was automatically loaded. The NanoHPLC (high performance liquid chromatography) system delivered solvents A (0.1% (vol/vol) formic acid) and B (99.9% (vol/vol) acetonitrile, 0.1% (vol/vol) formic acid) at a rate of 0.50 µL/min to load the peptides over a 30-min period and a rate of 0.3 µL/min to elute peptides directly into the nanoelectrospray. This was done with a gradual gradient from 0% (vol/vol) B to 30% (vol/vol) B over 150 min and concluded with a 10-min fast gradient from 30% (vol/vol) B to 50% (vol/vol) B at which time a 7-min flash-out from 50%-95% (vol/vol) B took place. As peptides eluted from the HPLC-column/electrospray source, survey MS scans were acquired in the Orbitrap with a resolution of 120,000 followed by CID-type (collision-induced dissociation) MS/MS fragmentation of the 30 most intense peptides detected in the MS1 scan from 350 to 1800 m/z. Redundancy was limited by dynamic exclusion.

#### Data analysis

Elite acquired MS/MS data files were converted to mgf file format using MSConvert (ProteoWizard) (76). For ATCC 14018, resulting mgf files were used to search against Uniprot *G. vaginalis* proteome databases (UP000001453, 10/2020 download, 1,365 total entries) along with a cRAP common lab contaminant database (116 total entries), whereas files for 2492 were used to search the *G. vaginalis* amino acid sequence database with a decoy reverse entries and a list of common contaminants (2,805 total entries). This was done using the in-house *Mascot* search engine 2.7.0 (Matrix Science) with fixed cysteine carbamidomethylation and variable methionine oxidation plus asparagine or glutamine deamidation. Peptide mass tolerance was set at 15 ppm and fragment mass at 0.6 Da. Protein annotations, significance of identification, and spectral-based quantification were done with Scaffold software (version 4.11.0, Proteome Software Inc., Portland, OR). For ATCC 14018, peptide identifications were accepted if they could be established at a greater than 94.0% probability to achieve an FDR (false discovery rate) less than 1.0% by the Scaffold Local FDR algorithm. Protein identifications were accepted if they could be established at greater than 98.0% probability to achieve an FDR less than 1.0% and contained at least two identified peptides. For 2492, peptide identifications were accepted if they could be established at greater than 97.0% probability to achieve an FDR less than 1.0% by the Scaffold Local FDR algorithm. Protein identifications were accepted if they could be established at greater than 99.0% probability to achieve an FDR less than 1.0% and contained at least two identified peptides. Protein probabilities were assigned by the Protein Prophet algorithm (77). Proteins that contained similar peptides and could not be differentiated based on MS/MS analysis alone were grouped to satisfy the principles of parsimony. For ATCC 14018, proteins sharing significant peptide evidence were grouped into clusters. Data are available on Dryad via https://doi.org/10.5061/dryad.zkh1893h0 and https://doi.org/10.5061/dryad.n8pk0p33f.

KEGG protein category assignments were made manually by searching for gene names in the *G. vaginalis* ATCC 14019 genome (gvg) within the KEGG GENES database (78). Proportions do not total to 1.0, as proteins could be classified into multiple categories.

### Human tissues

Human cervix samples were obtained from consented donors undergoing hysterectomies through the National Disease Research Interchange (NDRI). Donors were aged 50 years or younger and had not previously received chemotherapy or radiation treatment. Tissue specimens were examined by a pathologist for abnormalities and shipped overnight on ice in Dulbecco’s Modified Eagles Medium (DMEM) with penicillin and streptomycin. Upon arrival, endocervical and ectocervical regions were identified, separated, and processed into 3-mm biopsy punches. Tissue punches were allowed to recover at least overnight and maintained in cervical tissue culture medium (CTCM: CMRL-1600 medium, 0.1 µg/mL hydrocortisone 21-hemisuccinate sodium, 1 µg/mL bovine insulin, 2 mM L-glutamine, 5% heat-inactivated FBS) with penicillin and streptomycin at 37°C with 5% CO_2_ until experimentation (79). This work was determined to be exempt as human subjects research by the University of Wisconsin Health Sciences IRB as NDRI codes each tissue sample and does not label or provide identifying information.

### Cervix infections

Ectocervix punches were washed and moved to a medium without antibiotics. Bacteria were cultured on HBT agar overnight at 37°C with 5% CO_2_. They were collected from plates into PBS, washed in CTCM, and added at a final OD_600_ of 0.2 (~1 × 10^8^ CFU/mL) in 1 mL of CTCM, with or without cervix tissue, in a 24-well plate. Bacterial viability over time was assayed by serially diluting aliquots of medium and plating onto BHIFF agar. Plates were incubated at 37°C with 5% CO_2_ for 48 h, and colonies were counted to determine CFU/mL at each time point. Experiments were independently replicated 3–6 times.

### Genomic DNA extraction for sequencing

Strains AKK101 and 3336 were grown at 37°C anaerobically (5% CO_2_, 90% N_2_, 5% H_2_) on sBHI plates supplemented with 10% FBS. Bacterial pellets were lysed in colony lysis solution (1% Triton X-100, 2 mM EDTA (Ethylenediaminetetraacetic acid), 20 mM Tris-HCl) with a “pinch” of lysozyme, and the DNA was extracted using a DNeasy Blood & Tissue Kit (Qiagen). DNA quantity and quality were assessed via FEMTO Pulse at VCU Genomics Core. Strain 2492 was grown at 37°C with 5% CO_2_ in BHI_YDS_ medium for ~24 h. QIAGEN’s Genomic Tip Kit was used to extract high-quality, high-molecular weight DNA from bacterial pellets. The manufacturer’s protocol for the 20G tips was used with minor modifications. The quality of the extracted DNA was measured on a NanoDrop One instrument (ThermoFisher Scientific). Concentrations, 260/230 ratios, and 260/280 ratios were logged. Quantification of the extracted DNA was measured using the Qubit dsDNA High Sensitivity kit (ThermoFisher Scientific). Samples were diluted before running on the Agilent FemtoPulse System to assess DNA sizing and quality.

### Whole genome sequencing

#### Strains AKK101 and 3336

Whole-genome sequencing for strains AKK101 and 3336 was performed using the MiSeq platform (Illumina, San Diego, CA, USA) after the construction of a paired-end sequencing library. Sequencing depth was 700× for AKK101 Lg, 600× for AKK101 Sm, 400× for 3336 Lg, and 200× for 3336 Sm.

#### Strain 2492

A Pacific Biosciences HiFi library was prepared according to PN 101–853-100 Version 03 (Pacific Biosciences). Modifications include shearing with Covaris gTUBEs and size selection with Sage Sciences BluePippin. Library quality was assessed using the Agilent FemtoPulse System. Library was quantified using the Qubit dsDNA High Sensitivity kit. The library was sequenced on a Sequel II using Sequel Polymerase Binding Kit 2.2.

### Sequence assembly

#### Strains AKK101 and 3336

Raw sequencing data were assembled using a reference-guided assembly pipeline (https://github.com/myoungblom/RGAPepPipe_MAY). Quality of sequencing data was assessed using FastQC v0.8.11, and reads were trimmed using TrimGalore v0.6.4 (https://github.com/FelixKrueger/TrimGalore) (80). Reads were aligned using a reference sequence from the same clade using BWA MEM v0.7.12 (81, 82); reference 14018 GenBank accession AP012332.1 was used for strain AKK101 and reference N144 Genbank accession GCA_003408835.1 was used for strain 3336. Alignments were processed using samtools v1.3.1, and Picard v1.183 (http://broadinstitute.github.io/picard/) was used to remove duplicates and add read information. Genome assemblies were polished using Pilon v1.16, and quality was assessed using Qualimap BamQC (82, 83).

#### Strain 2492

Reads were assembled *de novo* using Flye v. 2.9-b1768 (84).

Sequencing data are available at BioProject PRJNA1082323 from NCBI.

### Sequence analysis

#### Strains AKK101 and 3336

SNPs were called using SNP sites v2.0.3 and annotated based on NCBI GenBank annotation and Prokka (71, 85).

#### Strain 2492

The assembled genome of the small colony morphotype was annotated using Prokka. Pbmm2 from PacBio tools was used to align small and large PacBio sequencing reads in BAM format to the small reference genome (https://github.com/PacificBiosciences/pbmm2). Allele frequency changes were calculated between the two aligned .bam files indexed to the small genome using Popoolation2 and in-house scripts (https://github.com/topfm/poolER) (86).

Homopolymer tracts (eight or more consecutive nucleotides) were searched, counted, and characterized manually using SnapGene Viewer. Strains (91) used for the analysis of homopolymer distribution across the *Gardnerella* genus are the same that were used in the phylogenetic tree.

### Homopolymer tract sequencing

Bacteria were first grown on HBT agar overnight at 37°C with 5% CO_2_ then restreaked onto BHIFF to isolate individual Lg colonies. After 2 days of growth on BHIFF at 37°C with 5% CO_2_, colonies were transferred to 50 µL colony lysis buffer (1% Triton X-100, 2 mM EDTA, 20 mM Tris-HCl, pH 8.5) and heated to 94°C for 15 min to yield PCR-ready template. The following primer pair, with an expected amplification region of 546 bp, was used for amplifying the homopolymer tract within the predicted GA module protein homolog in both strains via PCR: 5′-TTCGACCAAGAATCACCCAG-3′ and 5′-ACGCGAACAAACTTTCCAAG-3′. Amplification was performed in a 50 µL reaction volume with 25 µL Phusion Master Mix, 2.5 µL of each primer, 5 µL of colony lysate (template), and 15 µL of H_2_O. Cycling conditions were as follows: initial denaturation at 98°C for 30 sec, 35 cycles of 98°C for 5 sec, 63°C for 10 sec, and 72°C for 10 sec, with a final extension of 72°C for 5 min. DNA for each reaction was electrophoresed on a 0.8% agarose gel in TBE (Tris-borate-EDTA) buffer. The desired fragment size was cut from the gel, purified using a QIAquick Gel Extraction Kit (Qiagen), and sent to Functional Biosciences (Madison, WI) for Sanger sequencing.

### *ulaA* sequencing

The Sm colony phenotype of strain AKK101 was streaked from the freezer onto HBT and grown overnight at 37°C with 5% CO_2_. A single colony was restreaked onto BHIFF; a portion of the colony was transferred to colony lysis buffer as above to yield a parent colony template for PCR. Bacteria were incubated on BHIFF at 37°C with 5% CO_2_ for at least 2 days, and the lysate was prepared from daughter Sm and Lg colonies (three each) as above. The following primer pair, with an expected amplification region of 1,037 bp, was used for amplifying the region of *ulaA* in strain AKK101 containing mutations between colony variants: 5′-GAGAGAACTATGAACGGTGTTCTG-3′ and 5′-GTGCCAGCCAAAGCCATAATAATC-3′. Amplification was performed in a 50 µL reaction volume with 25 µL Phusion Master Mix, 2.5 µL of each primer, 1 µL of colony lysate (template), and 19 µL of H_2_O. Cycling conditions were as follows: initial denaturation at 98°C for 30 sec, 35 cycles of 98°C for 5 sec, 63.8°C for 10 sec, and 72°C for 10 sec, with a final extension of 72°C for 5 min. DNA for each reaction was electrophoresed on a 0.8% agarose gel in TBE buffer. The desired fragment size was cut from the gel, purified using a QIAquick Gel Extraction Kit (Qiagen), and sent to Functional Biosciences (Madison, WI) for Sanger sequencing.

### Statistics

All data were assessed for normality using the Shapiro-Wilk test. Equality of variance was assessed using the Brown-Forsythe test. The means of normally distributed data with equal variance were compared by using a Student’s *t* test. The means of normally distributed data with unequal variance were compared using Welch’s *t* test. The means of nonnormally distributed data were compared using a nonparametric Wilcoxon text with post hoc pairwise Wilcoxon tests, where applicable. All statistical analyses were performed using JMP Pro 17.0 software (SAS Institute Inc., Cary, NC, USA). *P* values of less than 0.05 were considered statistically significant.

## SUPPLEMENTAL MATERIAL

Supplemental material msphere.00450-24-s0001.pdf Supplemental figures and tables.

ASM does not own the copyrights to Supplemental Material that may be linked to, or accessed through, an article. The authors have granted ASM a non-exclusive, world-wide license to publish the Supplemental Material files. Please contact the corresponding author directly for reuse.
