# Supplementary material for: Phase variable colony variants are conserved across Gardnerella spp. and exhibit different virulence-associated phenotypes
Source: mSphere. 2024 Jun 27;9(7):e00450-24. doi: 10.1128/msphere.00450-24 (PMC11287997; doi:10.1128/msphere.00450-24)
Supplement: Supplemental material — Supplemental figures and tables. [file msphere.00450-24-s0001.pdf]

| Strain | Species | Vaneechoutte *et al.* species number | Clade | Op/T | Lg/Sm |
|---|---|---|---|---|---|
| AMD | *G. leopoldii* | 5 | 4/A | Y | Y |
| ATCC 14018 | *G. vaginalis* | 1 | 1/C | Y | Y |
| 101 | Unnamed | 8 | 3/D | Y | Y |
| JCP7275 | *G. vaginalis* | 1 | 1/C | Y | Y |
| **AKK101** | ***G. vaginalis*** | **1** | **1/C** | **Y** | **Y** |
| MH502 | Unnamed | 3 | 2/B | Y | Y |
| 805 | Unnamed | 8 | 3/D | Y | Y |
| **2492** | **Unknown** | **NA** | **NA** | **Y** | **Y** |
| 61/19V5 | Unnamed | 9 | 3/D | Y | Y |
| **3336** | **Unnamed** | **3** | **2/B** | **Y** | **Y** |
| UM035 | *G. piotii* | 4 | NA | Y | Y |
| 1400E | Unnamed | 2 | 1/C | Y | Y |
| 1500E | Unnamed | 10 | 3/D | Y | Y |
| A | *G. swidsinskii* | 6 | 4/A | Y | Y |
| B | *G. swidsinskii* | 6 | 4/A | Y | Y |

**Table S1. Various *Gardnerella* species exhibit both colony phenotypes.** The presence of both phenotypes was confirmed by streaking strains onto BHIFF (colony size) or sBHI agar plates supplemented with 10% FBS (opacity). Only four of the proposed thirteen Vaneechoutte *Gardnerella* species have been given a species name designation. The remaining nine are only recognized by their number. ”NA” means that a strain cannot be classified into one of the 13 Vaneechoutte species groups. These species designations were determined by *cpn60* sequence alignment (Fig. S1). ”Y” = yes, both phenotypes observed. Bolded strains are those characterized in this study.

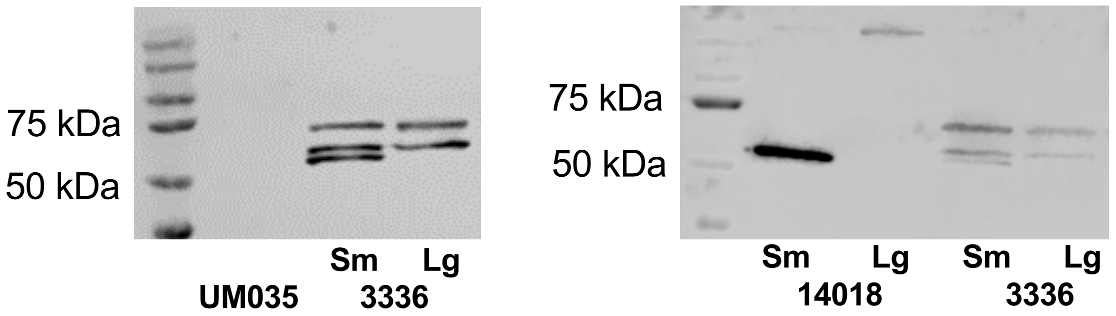


**Figure S1. Western blots of VLY in *Gardnerella* spp. supernatants.** Negative (left) and positive (right) control Western blots. Reported VLY molecular weight is 57 kDa.

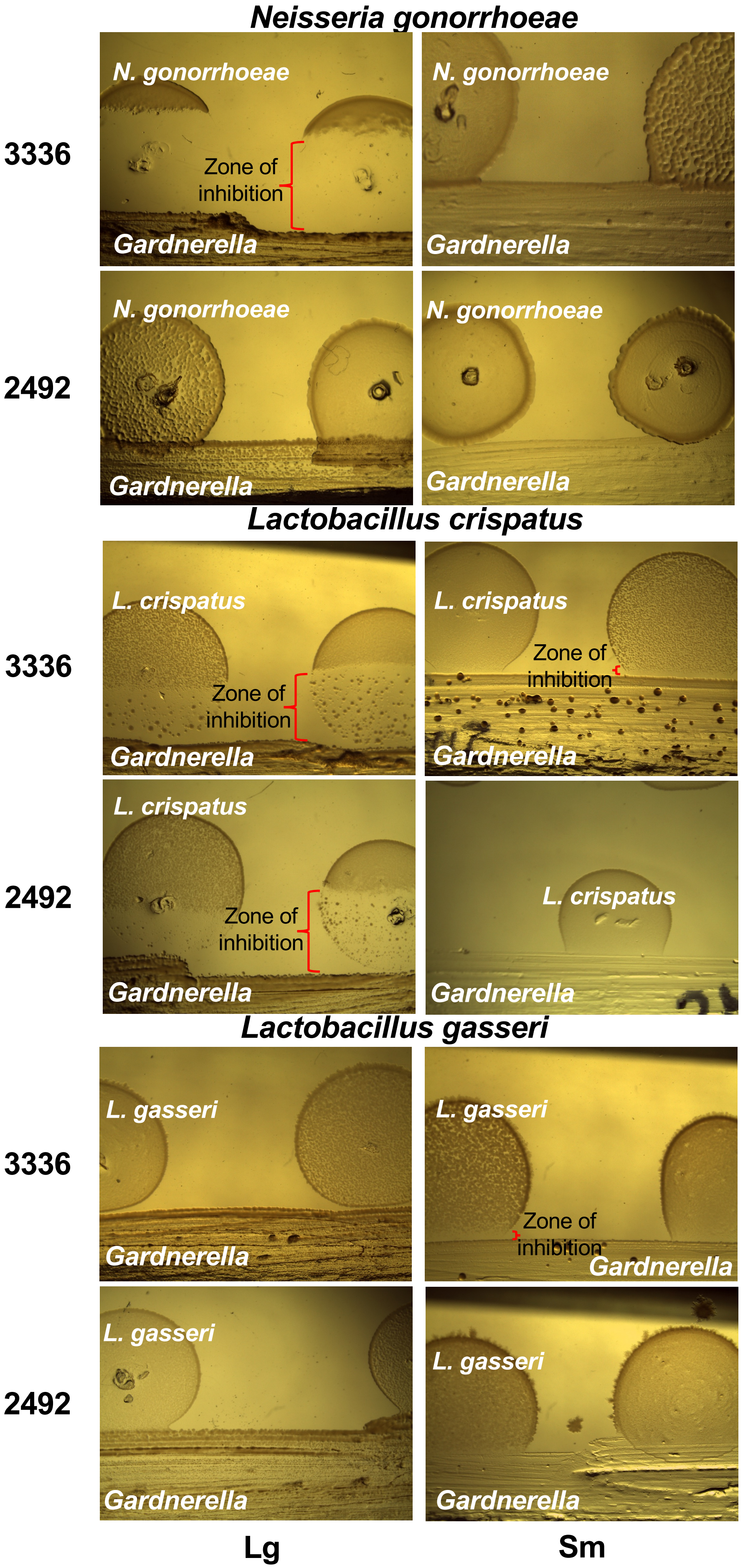


**Figure S2. Colony variants differ in their antagonism of vaginal bacteria.** *Gardnerella* colony variants were streaked onto $BHI_{YDS}$ + 10% FBS and grown for 24 hours before traversing the streak with 5µl of diluted *bacterial* cultures. Results from a representative experiment are shown but three independent experiments were performed. Images were taken on a Leica S8 APO stereo microscope.

| Protein annotation | Gene name | Fold difference in 2492 | Fold difference in ATCC 14018 |
|---|---|---|---|
| 10 kDa chaperonin | *groS* | 4.4 | 1.4 |
| 60 kDa chaperonin | *groL* | 2.5 | 1.7 |
| Cysteine synthase | *cysK* | 2.3 | 12 |
| Aminotransferase, class I/II | HMPREF0421_20226 | 4.9 | 5 |
| Tyrosine--tRNA ligase | *tyrS* | 2.7 | 1.4 |
| 50S ribosomal protein L17 | *rplQ* | 2.2 | 1.9 |

**Table S2. Proteins with greater expression in large variants**. Proteins with similar expression pattern in both 2492 and ATCC 14018.

| Protein Annotation | Gene name | Fold difference in 2492 | Fold difference in ATCC 14018 |
|---|---|---|---|
| Formate C-acetyltransferase | *pflB* | 5.1 | 2 |
| ABC transporter, ATP-binding protein | HMPREF0421_20105 | 2.5 | 1.4 |
| Thiol-activated cytolysin | *vly* | 95 | 3.3 |
| 4-alpha-glucanotransferase | *malQ* | 23.7 | 3.3 |
| ABC transporter, solute-binding protein | HMPREF0421_20232 | 3.9 | 1.4 |
| ABC transporter, ATP-binding protein | HMPREF0421_20436 | 4.1 | 2 |
| NADH oxidase | *noxE* | 8 | 1.3 |
| Phosphogluconate dehydrogenase (Decarboxylating) | *gnd* | 1.6 | 2 |
| Anaerobic ribonucleoside-triphosphate reductase | *nrdD* | 2.1 | 5 |
| RmuC domain protein | HMPREF0421_20072 | 2.1 | 2 |
| Phosphate-binding protein | *pstS* | 4.4 | 20 |
| Protein RecA | *recA* | 1.7 | 2 |
| Phosphate-specific transport system accessory protein | *phoU* | 4 | 1.7 |
| ABC transporter, permease protein | HMPREF0421_20240 | 2.7 | 3.3 |
| FtsK/SpoIIIE family protein | HMPREF0421_20871 | 2.5 | 5 |
| Pseudouridine synthase | HMPREF0421_20849 | 3.4 | 2 |
| Glucose-6-phosphate dehydrogenase assembly protein | HMPREF0421_21327 | 3.7 | 1.4 |
| Phosphoribosylformylglycinamidine synthase | HMPREF0421_20874 | 2.3 | 3.3 |
| Penicillin-insensitive transglycosylase | HMPREF0421_20394 | 2.1 | 5 |
| Branched-chain amino acid transport system carrier protein | *brnQ* | 2.4 | 3.3 |

**Table S3. Proteins with greater expression in small variants**. Proteins with similar expression pattern in both 2492 and ATCC 14018.

| Protein annotation | Gene name | Fold difference |
|---|---|---|
| Putative calcium-translocating P-type ATPase, PMCA-type | HMPREF0421_20193 | 18.5 |
| DNA topoisomerase (ATP-hydrolyzing) | HMPREF0421_20607 | 15.5 |
| Aminotransferase, class I/II | HMPREF0421_20226 | 4.9 |
| Ribonuclease J | *rnj* | 4.8 |
| 10 kDa chaperonin | *groS* | 4.4 |
| Peptidase C1-like family | HMPREF0421_20285 | 3.4 |
| Pyridine nucleotide-disulfide oxidoreductase | HMPREF0421_20163 | 3.4 |
| Glutamine synthetase | *glnA* | 3.1 |
| ABC transporter, substrate-binding protein | HMPREF0421_20686 | 2.8 |
| Tyrosine--tRNA ligase | *tyrS* | 2.7 |
| GTP-binding protein TypA | *typA* | 2.5 |
| 60 kDa chaperonin | *groL* | 2.45 |
| Ribonucleoside-diphosphate reductase | HMPREF0421_21332 | 2.45 |
| Ribose-phosphate pyrophosphokinase | *prs* | 2.5 |
| DNA-directed RNA polymerase subunit alpha | *rpoA* | 2.4 |

**Table S4. Proteomic differences between large and small colony variants.** The top 15 proteins with highest Lg/Sm ratio (fold difference) in strain 2492 are shown. Data are presented as an average of three independent experiments.

| Protein annotation | Gene name | Fold difference |
|---|---|---|
| Repeat protein (InlB) | HMPREF0421_21350 | 43 |
| Cysteine synthase | *cysK* | 12 |
| Alpha-L-fucosidase | HMPREF0421_20101 | 9.8 |
| ATP synthase, delta/epsilon subunit, beta-sandwich domain protein | HMPREF0421_20002 | 9.2 |
| ATP synthase subunit b | *atpF* | 8.4 |
| ABC transporter, solute-binding protein | HMPREF0421_20097 | 8.3 |
| DnaJ domain protein | HMPREF0421_20259 | 6.8 |
| Serine acetyltransferase | HMPREF0421_21032 | 5.6 |
| Ribokinase | *rbsK* | 5.4 |
| Argininosuccinate synthase | *argG* | 5.3 |
| ABC transporter, solute-binding protein | HMPREF0421_20451 | 5.2 |
| Aminotransferase, class I/II | HMPREF0421_20226 | 5 |
| ABC transporter, ATP-binding protein | HMPREF0421_21100 | 4.9 |
| Beta-galactosidase | HMPREF0421_20100 | 4.7 |
| SUF system FeS assembly protein, NifU family | HMPREF0421_20529 | 3.6 |

**Table S5. Proteomic differences between large and small colony variants.** The top 15 proteins with highest Lg/Sm ratio (fold difference) in strain ATCC 14018 are shown. Data are presented as an average of three independent experiments.

| Protein annotation | Gene name | Fold difference |
|---|---|---|
| Thiol-activated cytolysin | HMPREF0421_20066 | 95 |
| 4-alpha-glucanotransferase | *malQ* | 23.7 |
| Phosphomethylpyrimidine synthase | *thiC* | 15 |
| ABC transporter, ATP-binding protein | HMPREF0421_20038 | 11 |
| Ribosome hibernation promoting factor | *raiA* | 9.9 |
| NADH oxidase | *noxE* | 8 |
| 3-deoxy-7-phosphoheptulonate synthase | HMPREF0421_20347 | 8 |
| Hydrolase, alpha/beta domain protein | HMPREF0421_20703 | 7.3 |
| GA module protein | HMPREF0421_21196 | 7 |
| Oxidoreductase, aldo/keto reductase family protein | HMPREF0421_20336 | 5.3 |
| Formate C-acetyltransferase | *pflB* | 5.1 |
| ATPase family associated with various cellular activities (AAA) | HMPREF0421_20653 | 5 |
| Universal stress family protein | HMPREF0421_20544 | 4.7 |
| 2-dehydropantoate 2-reductase | HMPREF0421_20203 | 4.6 |
| ROK family protein | HMPREF0421_20797 | 4.5 |

**Table S6. Proteomic differences between large and small colony variants.** The top 15 proteins with highest Sm/Lg ratio (fold difference) in strain 2492 are shown. Data are presented as an average of three independent experiments

| Protein annotation | Gene name | Fold difference |
|---|---|---|
| 1,4-alpha-D-glucan glucanohydrolase | *pulA* | 50 |
| GA module-containing protein | HMPREF0421_20447 | 24 |
| Phosphate-binding protein PstS | *pstS* | 20 |
| ABC transporter, ATP-binding protein | HMPREF0421_21028 | 14.3 |
| L-serine ammonia-lyase | HMPREF0421_20456 | 12.5 |
| Phosphorylase, Pnp/Udp family | HMPREF0421_21030 | 12.5 |
| Non-specific serine/threonine protein kinase | HMPREF0421_20603 | 11.1 |
| ABC transporter, solute-binding protein | HMPREF0421_20246 | 10 |
| ABC transporter, permease protein | HMPREF0421_20235 | 10 |
| DNA primase | *dnaG* | 10 |
| Arylsulfatase | HMPREF0421_20878 | 10 |
| Peptidase, S9A/B/C family, catalytic domain protein | HMPREF0421_20392 | 10 |
| Sugar ABC transporter substrate-binding protein | HMPREF0421_20368 | 10 |
| Phosphate import ATP-binding protein PstB | *pstB* | 10 |
| Triosephosphate isomerase | *tpiA* | 10 |

**Table S7. Proteomic differences between large and small colony variants.** The top 15 proteins with highest Sm/Lg ratio (fold difference) in strain ATCC 14018 are shown. Data are presented as an average of three independent experiments

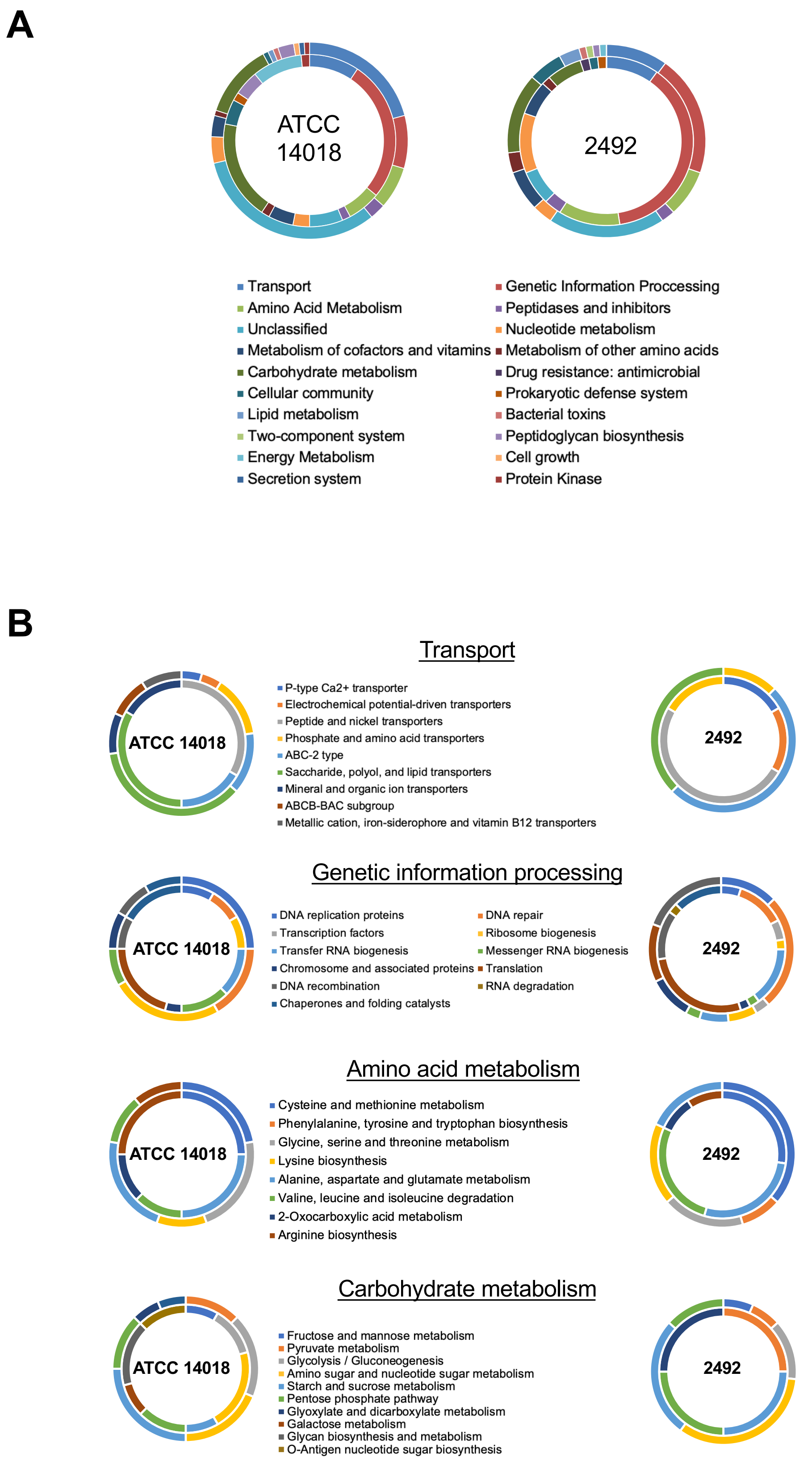


**Figure S3. Protein classes differ by variant and strain.** (A) Proteomics data grouped by KEGG protein class. (B) Subclass. Inner ring is the Lg variant, outer ring is the Sm variant.
